# Genome of a citrus rootstock and global DNA demethylation caused by heterografting

**DOI:** 10.1038/s41438-021-00505-2

**Published:** 2021-04-01

**Authors:** Yue Huang, Yuantao Xu, Xiaolin Jiang, Huiwen Yu, Huihui Jia, Chunming Tan, Gang Hu, Yibo Hu, Muhammad Junaid Rao, Xiuxin Deng, Qiang Xu

**Affiliations:** Key Laboratory of Horticultural Plant Biology (Ministry of Education), Huazhong Agricultural University, 430070 Wuhan, China

**Keywords:** Genomics, Epigenomics, Plant breeding

## Abstract

Grafting is an ancient technique used for plant propagation and improvement in horticultural crops for at least 1,500 years. Citrus plants, with a seed-to-seed cycle of 5–15 years, are among the fruit crops that were probably domesticated by grafting. *Poncirus trifoliata*, a widely used citrus rootstock, can promote early flowering, strengthen stress tolerance, and improve fruit quality via scion–rootstock interactions. Here, we report its genome assembly using PacBio sequencing. We obtained a final genome of 303 Mb with a contig N50 size of 1.17 Mb and annotated 25,680 protein-coding genes. DNA methylome and transcriptome analyses indicated that the strong adaptability of *P. trifoliata* is likely attributable to its special epigenetic modification and expression pattern of resistance-related genes. Heterografting by using sweet orange as scion and *P. trifoliata* as rootstock and autografting using sweet orange as both scion and rootstock were performed to investigate the genetic effects of the rootstock. Single-base methylome analysis indicated that *P. trifoliata* as a rootstock caused DNA demethylation and a reduction in 24-nt small RNAs (sRNAs) in scions compared to the level observed with autografting, implying the involvement of sRNA-mediated graft-transmissible epigenetic modifications in citrus grafting. Taken together, the assembled genome for the citrus rootstock and the analysis of graft-induced epigenetic modifications provide global insights into the genetic effects of rootstock–scion interactions and grafting biology.

## Introduction

**Fig. 1:**
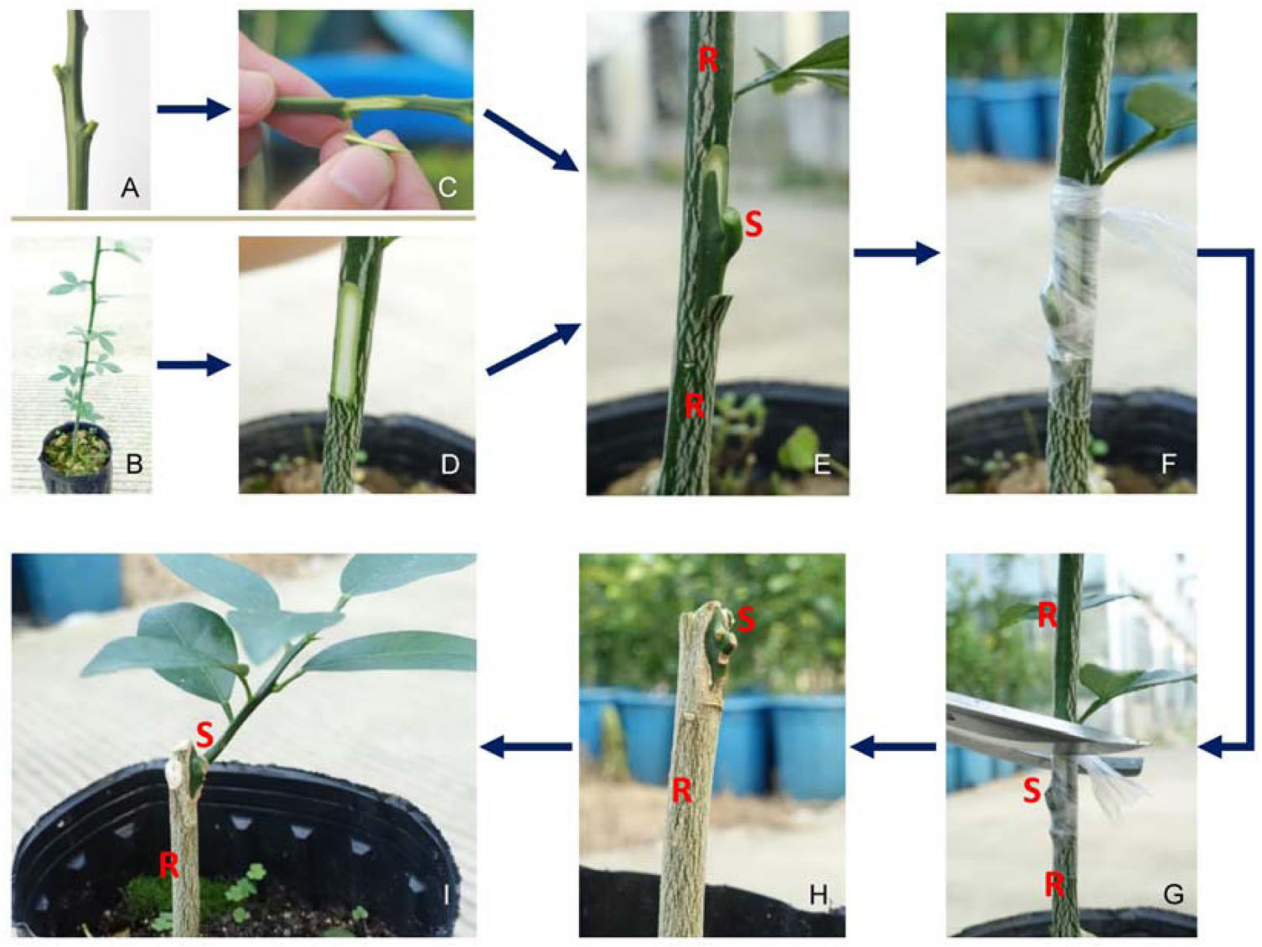
Schematic diagram of the citrus grafting process. **A** Mature sweet orange branch with full buds, which can be used as scion for grafting. **B** Annual seedling of *Poncirus trifoliata*, which can be used as rootstock for grafting. **C** Cutting the bud. **D** Cutting the rootstock. **E** Putting the bud on rootstock. **F** Binding the graft union with plastic film. **G** Cutting out the rootstock shoot above the graft union after graft union healing. **H** The live bud grafted on the rootstock. **I** New shoot coming out from the grafted bud. S scion, R rootstock

Plant grafting, as a traditional method of asexual propagation, is accomplished most commonly by connecting two plant segments, namely, a shoot piece called the ‘scion’ and a root piece known as the ‘rootstock’ (Fig. 1). This technique has been practiced in agriculture for over 2500 years^1^. Grafting has been widely used in modern production of many horticultural crops and some forest trees, such as citrus^2,3^, pear^4,5^, grape^6^, cassava^7^, and cedar^8^. Plant grafting is an ancient agricultural practice for propagation of uniform seedlings for commercial fruit species and to avoid a juvenile state, as an adult scion grafted onto a juvenile rootstock will maintain its adult state and ability to bear fruit^9^. Moreover, grafting can modulate plant growth^10^, improve the yield and quality of crops^11,12^, and enhance crop resistance to abiotic and biotic stresses^10,13–15^ in the form of scion–rootstock combinations.

Recently, increasing effort has been made to dissect the molecular and physiological mechanisms underlying grafting. The long-distance transport of signaling molecules, such as mobile proteins, mRNAs, small RNAs, and small molecules, between the scion and rootstock has been proven to play pivotal roles in grafting physiology^16^. Additionally, heterografting-induced DNA methylation polymorphisms have been detected in *Hevea brasiliensis*^17^, *Solanaceae* plants^18^, and *Cucurbitaceae* plants^19^. Some evidence also suggests that epigenetic modification of DNA methylation patterns may account for certain graft transformation phenomena^20–22^. Currently, advances in genomic resources and molecular techniques provide important opportunities for improving the understanding of scion–rootstock interactions.

Citrus crops are among the most important fruit tree crops in the world, with global production exceeding 147 million tons in 2017 (FAO, 2017). Due to their long juvenility (time to bearing) and high heterozygosity, citrus plants are generally reproduced by grafting to maintain the fine properties of the cultivar and reduce juvenility^23^. In the citrus industry, the use of suitable rootstocks plays a very important role in commercial citrus production. The rootstock has a significant impact on plant vigor, yield, fruit quality, and disease resistance^24–27^. Additionally, the rootstock can also affect the metabolome of citrus fruit juice, which determines the flavor and nutrition of the fruit^3,28^. Moreover, the rootstock can modulate the metabolic response to *Candidatus* Liberibacter asiaticus in grafted sweet orange^29^.

With a deeper understanding of the interaction between the rootstock and scion, the breeding of excellent citrus rootstocks is becoming one of the most important ways to improve the efficiency of citrus production and cope with the increasingly harsh planting environment and climate. In long-term citrus production practices, some suitable rootstocks have been widely used in different citrus growth regions, such as Troyer citrange, Carrizo citrange, and Swingle citrimelo in America^30^, Rangpur lemon in Brazil, sour orange in Italy and Mexico, Palestine sweet lemon and lime in Israel, and rough lemon in India^31^. The common advantages of these rootstock species are improved tree potential and enhanced tolerance to environmental stress or plant diseases. However, each rootstock still has some disadvantages that prevent it from meeting production demands. For example, Troyer citrange and Carrizo citrange are sensitive to *Citrus exocotis viroid*^32^, and the rough lemon as a rootstock produces poor fruit quality^33^. Therefore, breeding excellent rootstocks for the citrus industry is still ongoing.

*Poncirus trifoliata*, a wild species closely related to *Citrus* belonging to the Aurantioideae subfamily of the Rutaceae family, is a popular rootstock for the citrus industry in China. It is diploid and has the same number of chromosomes (2*n* = 18) as the *Citrus* genus^34^. It shows good grafting compatibility with most citrus varieties and exhibits favorable adaptation to a variety of environmental conditions, such as cold hardiness and tolerance to biotic stress factors, including the devastating Huanglongbing^35–37^. Poncirus seeds are highly polyembryonic and can produce uniform seedlings for ease of grafting and nursery management. Additionally, *P. trifoliata* is also a valuable parent for rootstock breeding because of its favorable characteristics. Crossing of *P. trifoliata* with orange gives Carrizo and Troyer citrange, which are used as the top commercial rootstocks in many citrus production areas^30^. Mining the excellent genetic resources of *P. trifoliata* and exploring rootstock–scion interactions may promote the improvement of citrus rootstocks and the development of the citrus industry.

In the present study, we aimed to understand the genetic basis of *P. trifoliata* as a citrus rootstock. We de novo assembled a high-quality genome of *P. trifoliata* by single-molecule sequencing, and whole-genome DNA methylation maps of *P. trifoliata* and sweet orange were drawn. Heterografting-induced changes in whole genome DNA methylation and sRNA abundance were evaluated. This study provides an important citrus rootstock genome for understanding the unique biology of grafting and should facilitate better application of grafting in the citrus industry.

## Results

### Citrus rootstock genome

Screening of 169 *P. trifoliata* accessions collected from Hubei, Henan, and Shanxi provinces in China indicated that the accession of ZK8 showed the lowest heterozygosity^38^ (Supplementary Table 1). This genotype was sequenced for genome assembly by using 91× coverage of third-generation long reads generated from the PacBio RS II platform (Supplementary Table 2). In addition, 38× Illumina-sequencing data were used to correct sequencing errors (Supplementary Fig. 1 and Supplementary Table 3). The sequence reads were assembled by Falcon^39^, resulting in 707 contigs. The total contig sequence length (303 Mb) covered 90.4% of the estimated *P. trifoliata* genome, and the contig N50 of the final assembly was 1.17 Mb (Table 1). To verify the quality of the assembly, the Illumina sequences were mapped to the assembled genome with a mapping rate of 96.6%, and the error rate of assembly was <0.01%, as estimated by the heterozygous SNP rate. BUSCO^40^ was also used to assess assembly completeness, and 97.4% of the eukaryotic gene sets were classified as complete. To construct pseudochromosomes, we mapped the contigs to 1934 markers with known sequences in a genetic linkage groups^41^, and 231 contigs (each >10 kb) were anchored, accounting for 231 Mb of the assembled *P. trifoliata* genome (Supplementary Table 4). Furthermore, 111 contigs that accounted for the majority of the length of the anchored scaffolds (164.5 Mb, or 71% of total anchored contigs) were in matched orientation within the genetic map, suggesting high alignment accordance between the anchored genetic markers and the sequence contigs.

**Table 1:** Statistics for the genome assembly of *Poncirus trifoliata*

Assembly feature	Statistic
Estimated genome size (by k-mer analysis)	335 Mb
Assembled size	303 Mb
Chromosome size	231 Mb
Number of contigs	707
Contig N50	1.17 Mb
Contig N90	189 kb
TE percentage	46.5
Annotated protein-coding genes	25,680
Average coding sequence length	1297 bp
Average exons per gene	6.4

**Fig. 2:**
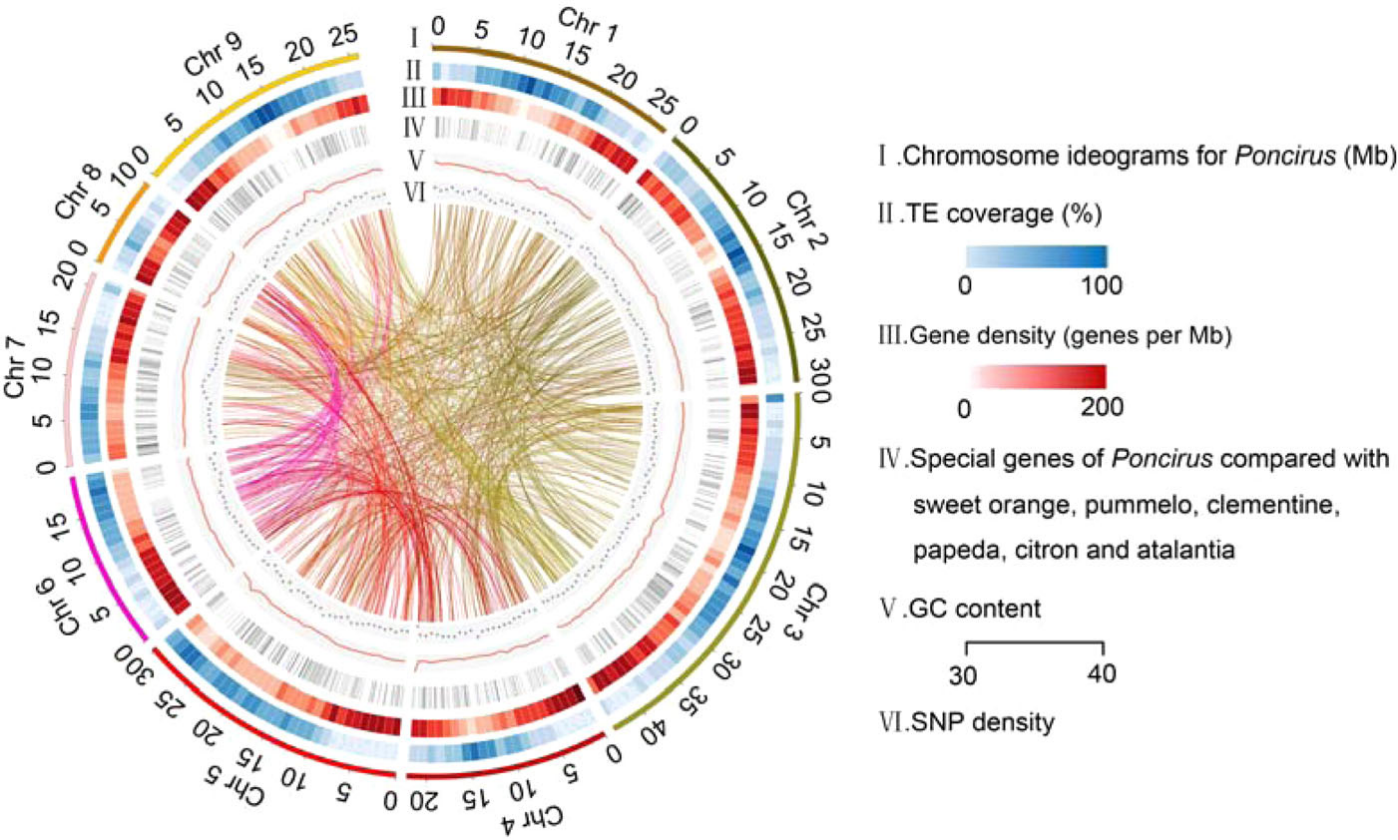
Characterization of the *Poncirus trifoliata* genome. The designation of each track is listed on the right. The lines in the center of the circle indicate pairs of homologous genes on the different chromosomes of *P. trifoliata*

Ab initio gene predictions, homology searches, and RNA-seq analysis were integrated to predict gene models. In total, 25,680 genes with 39,675 transcripts were identified, with an average coding sequence length of 1297 bp and an average of 6.4 exons per gene. In addition, more than 99% of the protein-coding genes could be functionally annotated by GO terms, motifs, domains, and associated pathways. On the basis of homology searches and de novo methods, we identified a total of 140.9 Mb of repetitive elements, representing 46.5% of the genomic assembly (Table 1). Among the repetitive sequences, long terminal retrotransposons (LTRs) were the most abundant, accounting for 23.9% of the assembly (Supplementary Table 5). An overview of the gene density, repetitive elements, SNPs, and all detected syntenic blocks is presented in Fig. 2.

### Genome comparison among Poncirus and other Citrinae genomes

**Fig. 3:**
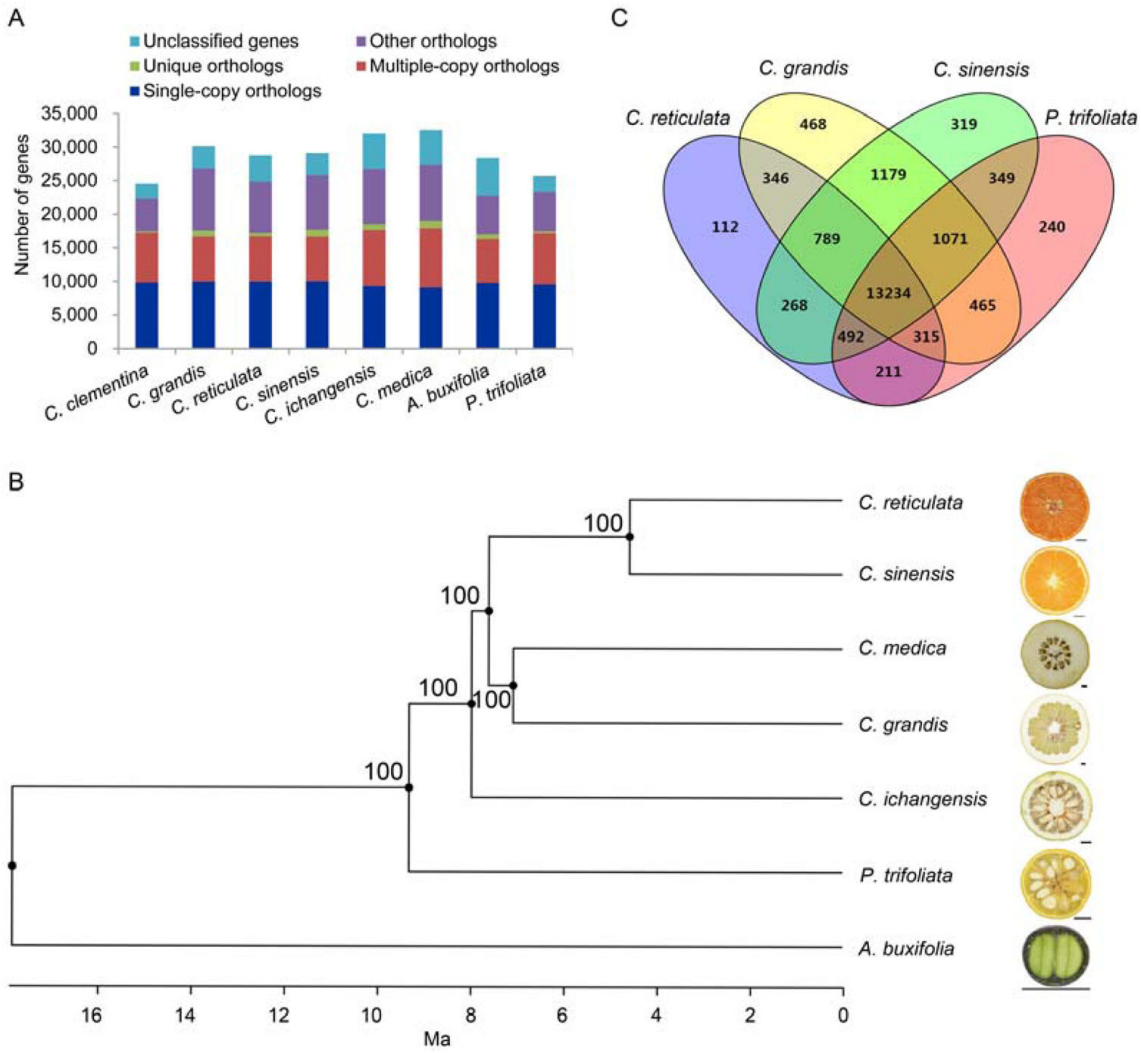
Protein orthology comparison among genomes of eight Citrinae species. **A** Clusters of orthologous and paralogous gene families in the eight Citrinae species identified by OrthoMCL. **B** Phylogenetic status of *P. trifoliata* in the Citrinae group. A maximum-likelihood phylogenetic tree was constructed by using all single-copy orthologs shared by the eight Citrinae species. The numbers near tree nodes are bootstrap values. Divergence times are indicated by bars below the phylogenetic tree scaled as million years ago (Ma). **C** Overlap of gene families in *P. trifoliata* and three widely cultivated citrus species

Pairwise comparisons of putative orthologs and paralogs were analyzed among *P. trifoliata* and seven other Citrinae group members, including *Citrus grandis*, *Citrus reticulata*, *Citrus sinensis*, *Citrus clementina*, *Citrus medica*, *Citrus ichangensis*, and *Atalantia buxifolia* (Fig. 3A and Supplementary Table 6)^42–45^. Based on the analysis of gene family clustering, we identified 25,888 gene families, of which 11,860 were shared by all eight species, and 7261 of these shared families were single-copy gene families. The sequences of these single-copy orthologous genes were retrieved from the eight Citrinae species, and alignments were performed based on these sequences. We combined all the alignments to produce an alignment matrix for the construction of a phylogenetic tree (Fig. 3B). *P. trifoliata* is located between *Atalantia* and the cultivated citrus species, indicating its closer relationship to the cultivated citrus species than to *Atalantia*. These results also support the better grafting and sexual compatibility of *P. trifoliata* with cultivated citrus species than with *Atalantia*^46,47^.

To gain clues regarding the genes specific to *P. trifoliata*, we compared the gene families among *P. trifoliata* and three widely cultivated species. As shown in Fig. 3C, 240 gene families were specific to *P. trifoliata*, and 13,234 gene families were shared by *P. trifoliata*, *C. grandis*, *C. sinensis*, and *C. clementina*. GO studies based on the 240 *P. trifoliata-*specific gene families showed enrichment of genes encoding “multicellular organismal homeostasis”, “response to temperature stimulus”, and “tachykinin receptor signaling pathway”, suggesting that some of these genes may be related to the special cold resistance of *P. trifoliata* (Supplementary Table 7).

### Gene expression and DNA methylation variation between roots and shoots of *P. trifoliata*

To dissect the transcriptomic characteristics of *P. trifoliata* roots, we collected roots (representative of underground tissue) and shoots (representative of aboveground tissue) from 2-month-old seedlings of *P. trifoliata* and sweet orange to isolate RNA for transcriptome sequencing. The shoot and root transcriptomes were compared within each species, namely, *P. trifoliata* and sweet orange, separately. In sweet orange, the number of genes highly expressed in shoots (4057) was larger than that in roots (3581). In *P. trifoliata*, more genes were highly expressed in roots (3372) than in shoots (3290), suggesting that transcriptomic events are more frequent in the roots of *P. trifoliata* than in those of sweet orange. GO analysis indicated that the genes highly expressed in the roots of *P. trifoliata* and sweet orange were both significantly enriched (*P*-value < 0.05, FDR < 0.05) in secondary metabolic process, phenylpropanoid metabolic process, and regulation of root development (Supplementary Tables 8 and 9). Notably, we found that the genes highly expressed in *P. trifoliata* roots were specifically enriched (*P*-value < 0.05, FDR < 0.05) in 34 GO categories, including killing of cells of another organism, response to chitin, response to fungus, and defense response to oomycetes (Fig. 3A and Supplementary Table 8). These enriched genes may contribute to the strong environmental adaptability of *P. trifoliata* as a rootstock.

**Fig. 4:**
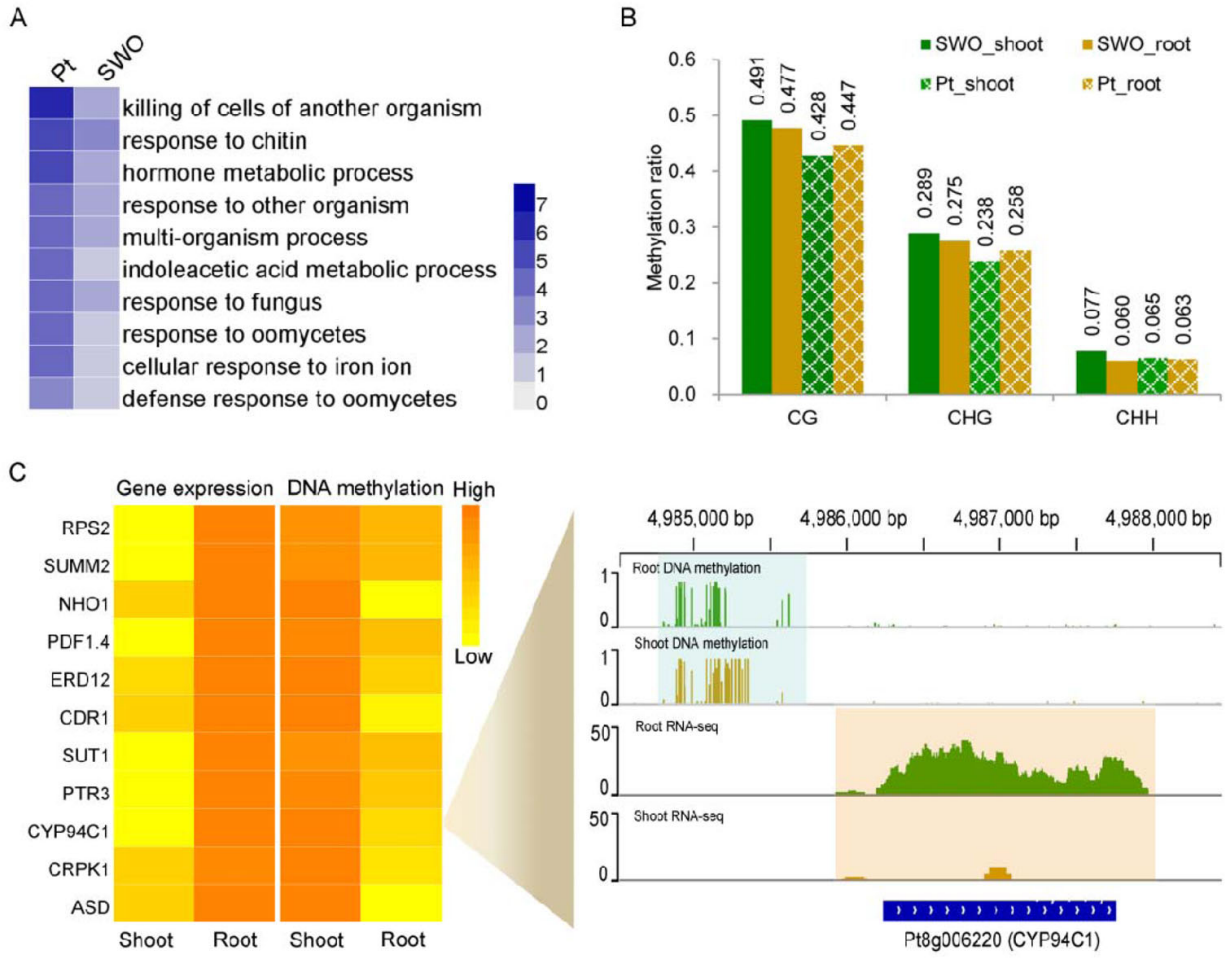
Differentially expressed genes and DNA methylation variation between roots and shoots. **A** Selected GO terms specifically enriched for the highly expressed genes in *P. trifoliata* roots. A darker blue color indicates a more significant *P*-value. All GO terms have a *P*-value < 0.05. **B** DNA methylation levels in the shoots and roots of *P. trifoliata* and sweet orange. **C** Heatmaps showing the expression levels and promoter methylation levels of selected resistance-related genes. The genome browser snapshot in the panel on the right shows the methylation level and expression level of *CYP94C1* in each developmental stage. The differentially methylated regions and expressed transcripts are shadowed with green and yellow boxes, respectively

In citrus, DNA methylation is dynamic and shows tissue specificity^48^. To investigate the DNA methylation changes between roots and shoots, whole-genome bisulfite sequencing was performed on the same set of materials used for transcriptome analysis. In total, 282,798,908, 291,934,320, 260,251,696, and 242,702,062 raw reads were generated for the roots of *P. trifoliata* (Pt_root), shoots of *P. trifoliata* (Pt_shoot), roots of sweet orange (SWO_root), and shoots of sweet orange (SWO_shoot), respectively (Supplementary Fig. 2 and Supplementary Table 10). The genome sequences of *P. trifoliata* and sweet orange were used as the reference for data analysis separately. Approximately 80% of cytosines were covered by at least one uniquely mapped read. Overall, the DNA methylation level of sweet orange was higher than that of *P. trifoliata* in the CG and CHG contexts (Fig. 4B). In *P. trifoliata*, the CG and CHG methylation levels in roots were slightly higher than those in shoots, while the opposite situation was observed in sweet orange (Fig. 4B). On each chromosome, methylcytosine densities in all contexts were not evenly spread, and DNA methylation was enriched predominantly in the pericentromeric regions (Supplementary Fig. 3). A chromosome-scale view of the DNA methylation levels and the densities of genes and transposable elements (TEs) showed that DNA methylation sites were most likely enriched in the regions containing numerous TEs but few genes. Similarly, the relative abundance of sRNAs was plotted along each chromosome, which showed a higher density of sRNAs in TE-rich regions, suggesting a role for these sRNAs in TE methylation (Supplementary Fig. 3).

To further evaluate the genes affected by DNA methylation, we identified differentially methylated regions (DMRs) between Pt_root and Pt_shoot. In total, we identified 13,331 high-confidence (FDR < 0.01) DMRs in all contexts. Regarding the DMR-associated genes, we identified 1705 genes that contained DMRs in their promoter region—1097 genes showed increased amounts of DNA methylation in roots, whereas the remaining 608 genes showed increased amounts of DNA methylation in shoots. Among the DMR-associated genes, we identified some genes with important roles in the defense response, such as *CDR1*, *RPS2*, and *PDF1.4*. The expression levels of these genes were upregulated in the roots of *P. trifoliata*, which may be associated with DNA hypomethylation occurring in their promoter regions (Fig. 4C).

### *P. trifoliata* as a rootstock induced DNA demethylation in the scion

**Fig. 5:**
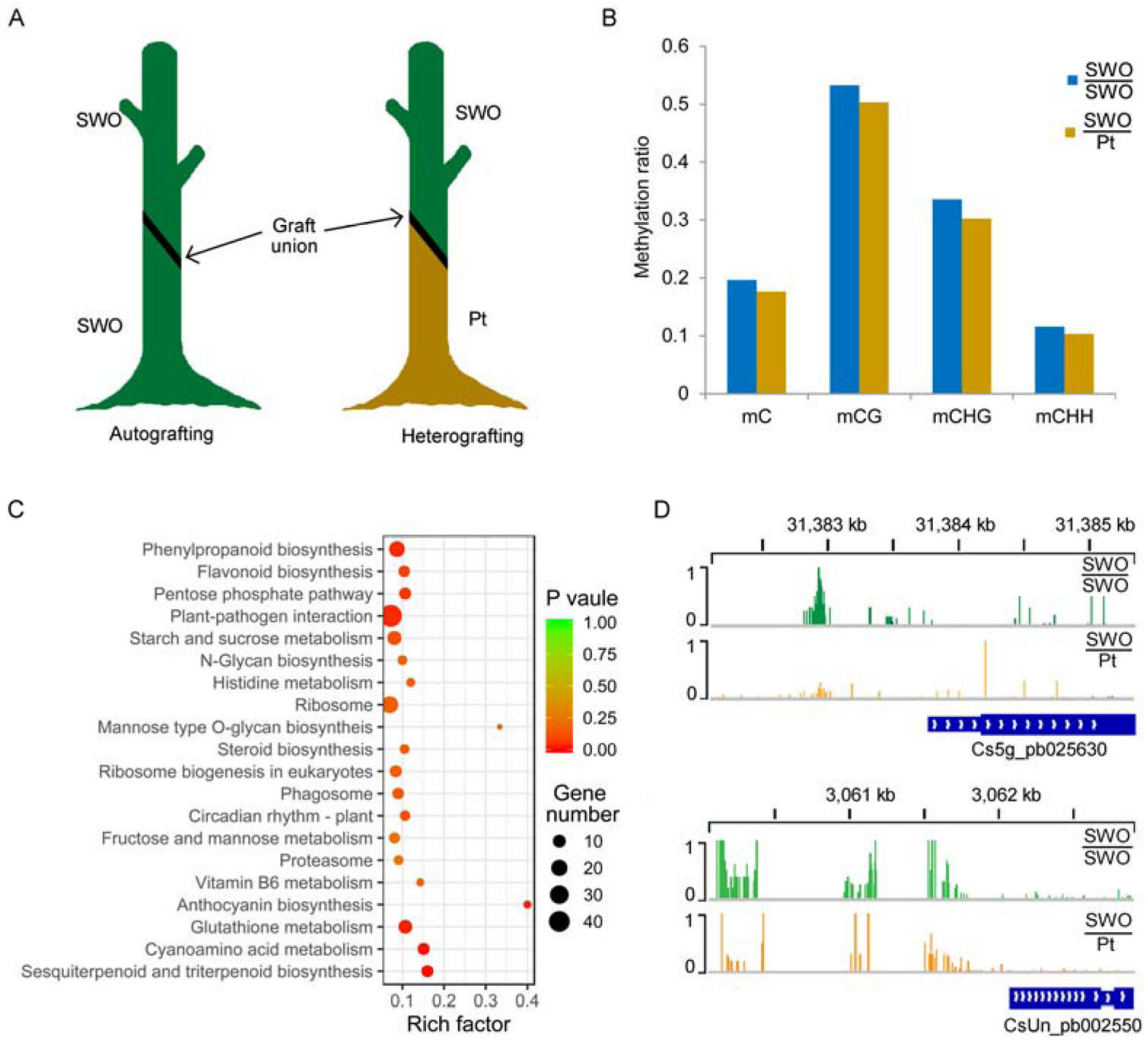
Poncirus as a rootstock induced DNA demethylation in the scion. **A** Schematic diagram showing autografting (SWO/SWO) and heterografting (SWO/Pt). SWO, sweet orange; Pt *Poncirus trifoliata*. The black lines represent the graft junction. **B** Genome-wide methylation levels in scions of two grafting combinations. **C** KEGG pathway-enrichment analysis of differentially methylated genes (DMGs) between scions of heterografted and autografted plants. **D** Genome browser snapshot showing DNA methylation levels of two disease resistance-related genes (*Cs5g_pb025630* and *CsUn_pb002550*) in scions of two grafting combinations

Recent studies have suggested that modifications of DNA methylation may be an important cause of grafting-induced variations^18,19,21^. Considering the special DNA methylation pattern observed in the root of *P. trifoliata* as mentioned above, we conducted whole-genome bisulfite sequencing to explore DNA methylation variation between leaves of sweet orange autografted on sweet orange (SWO/SWO) and heterografted on *P. trifoliata* (SWO/Pt) (Fig. 5A). In total, an average of 158,550,092 reads were generated for each replicate, yielding ~23.8 Gb of data representing >60× of the sweet orange genome (Supplementary Fig. 4 and Supplementary Table 11). Whole-genome methylation level comparison revealed that the levels of CG and CHG methylation both decreased by ~3% at the whole-genome level in the heterografting combination relative to the autografted plant (Fig. 5B), suggesting that demethylation was caused by heterografting. We examined the expression of all the DNA methylase genes (*MET*, *CMT2*, *CMT3*, *DRM*, and *DDM*) and demethylase genes (*ROS1*, *DME*, and *DML3*) by q-PCR. The expression of the DNA methylase genes showed no significant difference between SWO/SWO and SWO/Pt, except the expression of *CMT2*. The three DNA demethylase genes (*ROS1*, *DME*, and *DML3*) were upregulated when *P. trifoliata* was used as rootstock, which is consistent with hypomethylation being detected when *P. trifoliata* was used as rootstock (Supplementary Fig. 5). We investigated DNA methylation patterns throughout the gene and TE regions in scions of SWO/SWO and SWO/Pt and found that both grafting combinations showed similar patterns of CG, CHG, and CHH methylation in gene regions (Supplementary Fig. 6). In the TE and flanking regions, the methylation levels of all three contexts in the scion of SWO/Pt were ~2% lower than those in the scion of SWO/SWO (Supplementary Fig. 6), possibly accounting for the major DNA methylation difference caused by heterografting.

To further investigate changes in DNA methylation, DMRs were identified between SWO/SWO and SWO/Pt. In total, we identified 9027 DMRs in SWO/Pt compared to SWO/SWO, among which 4464 were hypermethylated and 4563 were hypomethylated. To assess how DNA methylation contributes to rootstock–scion interactions, 1537 genes that contained DMRs in their promoter region were identified (Supplementary Table 12). Kyoto Encyclopedia of Genes and Genomes (KEGG) pathway analysis of these genes revealed that many distinctive biological pathways were affected, such as phenylpropanoid biosynthesis, flavonoid biosynthesis, pentose phosphate pathway, and plant–pathogen interaction (Fig. 5C and Supplementary Table 13). Notably, we found 47 genes with DMRs in their promoter region that were related to plant–pathogen interaction, such as the disease resistance genes *RPP8* and *RPS2* and the transcription factors *MYB108* and *MYB44* (Fig. 5D and Supplementary Table 13). Additionally, some genes involved in these biological pathways were upregulated in SWO/Pt (Supplementary Table 14). These results indicated that heterografting-induced DNA methylation variation may be responsible for the strong adaptability of citrus cultivars when using *P. trifoliata* as rootstock.

### Heterografting reduced root-to-shoot mobile sRNAs

**Fig. 6:**
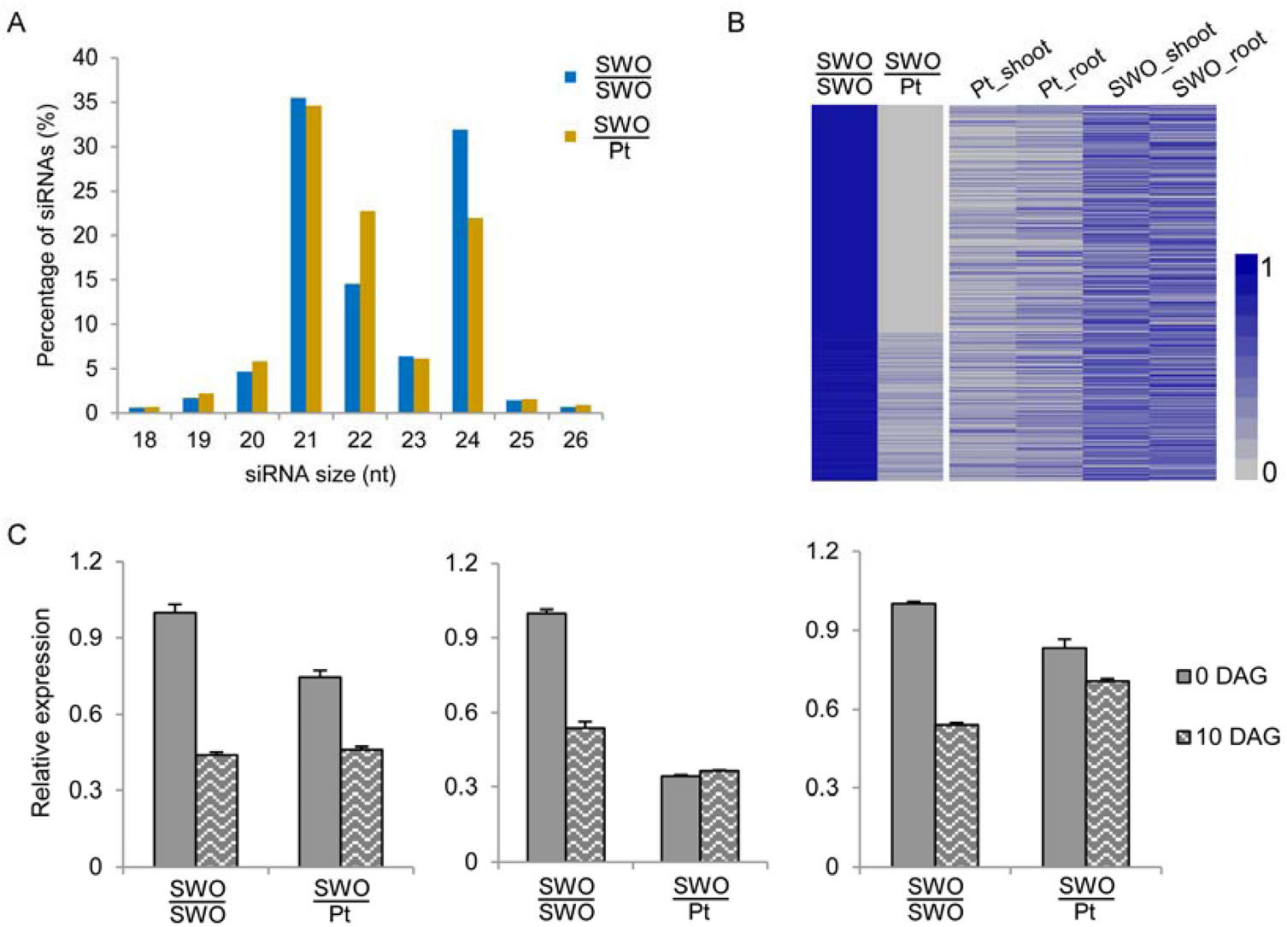
Heterografting reduced root-to-shoot mobile sRNAs. **A** Percentages of unique sRNAs in scions of two grafting combinations. **B** Heatmaps showing the expression pattern of DNA demethylation-associated 24-nt sRNAs in scions of two grafting combinations of roots and shoots of sweet orange and *P. trifoliata*. **C** qPCR detection of the expression levels of three selected 24-nt sRNAs in scions of the two grafting combinations before and 10 days after girdling (DAG). The three sRNAs from left to right are AAATGGATTAGGTATCCCATACCT, ATACCAACATTCTTTTCCAAGATT, and AACTATTACGCCTATTGAGCGATC. The values in each column are the means of three biological replicates. Error bars indicate the SD

To test the link between changes in DNA methylation and small RNAs, we generated small RNA-seq data from SWO/SWO and SWO/Pt leaves (Supplementary Table 15). The data showed that the percentage of 24-nt sRNAs was much lower in SWO/Pt than in SWO/SWO, which was consistent with the trend of DNA demethylation caused by heterografting (Fig. 6A, Supplementary Fig. 7A). Furthermore, we found that the methylation level of the regions overlapping with sRNAs was significantly higher than that of random genomic regions, suggesting that heterografting-induced DNA demethylation was significantly associated with sRNAs (*P* < 0.01) (Supplementary Fig. 7B).

The decreased 24-nt sRNAs in the scion of heterografted plants could be caused by repression of the sRNA biogenesis pathway or asymmetric movement of sRNAs between the rootstock and scion. To investigate these two possibilities, we first examined the expression levels of the key genes involved in 24-nt sRNA biogenesis in the scions of SWO/SWO and SWO/Pt. For the canonical RNA-directed DNA methylation (RdDM) pathway in *Arabidopsis*, five key genes are involved in 24-nt sRNA biogenesis, namely, *SHH1*, *NRPD1*, *CLSY1*, *RDR2*, and *DCL3*^49^. In the sweet orange genome, we identified the orthologs of these genes using BLASTP and found that none of the five genes were differentially expressed between the scions of SWO/SWO and SWO/Pt (Supplementary Fig. 8), indicating that the decreased 24-nt sRNAs in the scion of heterografted plants cannot be attributed to sRNA biogenesis. Then, we analyzed the expression levels (miRNA read counts were normalized to reads per million, RPM) of all the detected 24-nt sRNAs and the DNA methylation levels of their target loci. Among the 24-nt sRNAs with reduced DNA methylation in their target loci in the scion of SWO/Pt, 548 were SWO/SWO specific (RPM > 0 in SWO/SWO, not detected in SWO/Pt) and 356 were highly expressed in SWO/SWO (≥1.5-fold) (Table 2 and Supplementary Table 16). Further investigation of these 903 24-nt sRNAs in the roots of sweet orange and *P. trifoliata* revealed 534 24-nt sRNAs that were highly (≥1.5-fold) expressed in sweet orange roots (Fig. 6B). Therefore, we speculate that the higher abundance of the 24-nt sRNAs in sweet orange roots may have led to more 24-nt sRNAs moving to the scion when sweet orange was used as a rootstock for grafting. To verify the root-to-shoot movement of the 24-nt sRNAs, girdling treatment above the grafting union was applied for the two grafting combinations. qRT-PCR analyses of three selected 24-nt sRNAs revealed that in the scion of SWO/Pt, none of the 24-nt sRNAs showed significant changes in transcript levels before and after girdling (Fig. 6C). In the scion of SWO/SWO, the expression levels of the 24-nt sRNAs were obviously reduced after girdling (Fig. 6C), indicating that the phloem-mobile 24-nt sRNAs from roots to shoots were blocked by girdling.

**Table 2:** Expression of decreased 24-nt sRNAs in the scion of the heterografted plant and methylation level of the matched DMRs

siRNA sequence	Expression level		Methylation level	
	SWO/SWO	SWO/Pt	SWO/SWO	SWO/Pt
TGAGATAGTGGGGGAGCCTGGGTC	1.10	0.08	0.33	0.13
TACTCCGTGACTTCTTAAGTCGGT	1.02	0	0.28	0.13
GATCTACAAGAGAAAAAGAGGAGT	0.90	0.05	0.36	0.15
TAGCAATAAGCTGGTTGGGGGGTG	0.85	0.10	0.27	0.12
TTGGTTCCGGTGGAACATCCACAC	0.81	0	0.48	0.23
GGTCACATCATTTCGAGCAAAGGT	0.76	0	0.33	0.15
ACGATCCTGTTGGGCTAAAATTCT	0.75	0	0.38	0.14
TTTGTGGCTGTATCATTACTCTTT	0.75	0.05	0.27	0.04
TTGACTTTTGAAGTTTGACCAGCC	0.70	0.07	0.16	0.06
CTTTAAAATGCAAAGACCCAGGCT	0.69	0	0.33	0.13
TGCCACGTCACCATCAACAGTACA	0.59	0.04	0.33	0.16
AGATACCCAAGTACGTCATTTCAA	0.57	0.04	0.46	0.22
AACACACGCTTTTCTCCCCAAATT	0.54	0.04	0.42	0.18
TTTTCAAGGTACGATTTCTAGAAT	0.54	0.05	0.61	0.29
GAGACGACTAACTCTTCTCCAGTC	0.52	0	0.41	0.14
GGCTTCATACCCGGGCCGGGTAAT	0.51	0	0.32	0.15
AATGAGCTCCACGCCTGCAAAACA	0.51	0	0.26	0.08
GCTTGATGACATGACCAGTGTTCT	0.49	0	0.25	0.09
TCTAATGATCATTTTCAACATACT	0.48	0	0.81	0.52
CTGCTAATGAGCTACTTGATATGT	0.46	0.07	0.29	0.13

## Discussion

*P. trifoliata* is a Chinese deciduous species with trifoliolate leaves that has been used as a rootstock for citrus cultivars for a long time. It is resistant to Phytophthora, nematodes, and tristeza virus and can withstand a cold temperature of −26 °C^50,51^. Ancient people observed that the graft union of mandarin and *P. trifoliata* grown in South China was mandarin, while in North China, the graft union grown was trifoliate orange. This is now easy to explain, as only the rootstock trifoliate orange is highly resistant to cold temperatures and can survive in the winter in North China, while the scion mandarin with good fruit quality is vulnerable to cold temperatures. *P. trifoliata* is also sexually compatible with the *Citrus* genus, and the most widely grown sexual hybrid is Troyer citrange, which is also a very important citrus rootstock in many countries^23^. In this study, we sequenced and assembled a high-quality genome of a landrace of *P. trifoliata* from Shanxi Province based on genetic evaluation of a set of 169 accessions. Thus, this genotype represents an original type of *P. trifoliata* relative to the narrow genetic background in the United States; moreover, this genome is more complete than the recently published genome^52^ (Supplementary Table 6). Both genomes provide valuable genomic resource for citrus rootstock genetics and breeding improvement. This genome together with the genomes of cultivated citrus plants (as scions) should facilitate a deep understanding of scion–rootstock interactions at the genomic level. In addition, the genomic information may also be valuable for further investigation of the basis of medical uses and some other unique biological traits of trifoliate orange, such as cold hardiness, trifoliate leaf character, and resistance to citrus tristeza virus.

Grafting has been widely used to improve the performance of horticultural plants for thousands of years. Although increasing physiological evidence indicates the existence of rootstock–scion interactions in plants^53^, molecular evidence at the genetic and epigenetic levels revealing the influence of rootstock–scion interactions is scarce. Recently, the discovery of mobile genetic elements such as DNA, RNA, and proteins has gradually revealed the molecular mechanisms underlying several agronomic traits affected by rootstock–scion interactions^16^. For example, microRNA399 was identified as a long-distance signal for the regulation of plant phosphate homeostasis in rapeseed and pumpkin^54^. Epigenetic modification may also play important roles in creating heritable phenotypic variation by grafting. In this study, we found that the DNA methylation level in the scion grafted on *P. trifoliata* decreased by ~3% (Fig. 5). A previous study reported that DNA methylation levels in grafted cucumber and melon were significantly increased when pumpkin was used as rootstock, while there was no significant change in grafted watermelon^19^. Grafting between plant species of *Solanaceae* caused extensive DNA methylation variation, and some variation could be stably passed on to offspring^18^. Thus, we can conclude that grafting does cause variations in DNA methylation, but the degree and trend of DNA methylation variation may vary based on species and graft combinations. The effect of grafting on DNA methylation may be a regulatory mechanism for intercell interactions between scions and rootstocks^21^.

Our observation that the DNA demethylation pattern in the scions of heterografted plants is concomitant with reduced abundance of 24-nt sRNAs implies a possible mechanism of graft-induced alteration in DNA methylation. sRNA-mediated graft-transmissible epigenetic modifications have been confirmed in *Arabidopsis thaliana* by grafting experiments. Molnar et al. demonstrated that the movement of transgene-derived and endogenous sRNAs from shoot to root across graft unions can cause epigenetic changes in rootstock cells^55^. A subsequent study showed that mobile sRNAs originating in the shoots guided RdDM at thousands of loci in the roots^56^. Our finding that the sRNAs are associated with the methylation levels of the sRNA-overlapping regions suggests that the DNA demethylation in the scion of heterografted plants may be attributed to the reduction in sRNAs leading to reduced RdDM. As the genotypes of both the rootstock and scion in autografted plants are the same, it is difficult to determine whether the highly expressed sRNAs in the scions of autografted plants are from the rootstock. However, the higher expression of the sRNAs in the roots of sweet orange than in those of Poncirus, combined with the downregulation of the highly expressed sRNAs in the scions of autografted plants after griding treatment, suggested the possibility that the reduction in the rootstock-to-scion movement of sRNAs leads to decreased sRNA abundance in the scions of heterografted plants. Based on the above finding, the DNA demethylation in the scion of SWO/Pt was possibly caused by the reduced graft-transmissible epigenetic modifications mediated by rootstock-to-scion movement of sRNAs.

The high-quality citrus rootstock genome provides an important basis for future studies on rootstock genetic improvement, and our multiomic analysis of *P. trifoliata* may promote a deeper understanding of graft biology, not only in citrus plants but also in other horticultural crops. Given the important biological roles played by DNA methylation in plants, it is reasonable to suspect that graft-transmissible epigenetic modifications may have functional consequences. In the future, the use of transgenic plants as rootstocks for grafting will further enhance the opportunity to improve practical nontransgenic cultivars in the field.

## Methods

### Plant materials and sequencing

The *P. trifoliata* used for genome sequencing was collected from Hanzhong, Shanxi Province, China. For whole-genome bisulfite sequencing, the seeds of sweet orange and *P. trifoliata* were germinated and cultured in an artificial climate incubator (28 °C, 16 h light and 8 h darkness) for 2 months, and then, the shoots and roots of the seedlings were collected separately. Two biological repeats were performed for each tissue, and 8–10 seedlings were mixed for each biological repeat. The same materials were used to perform RNA and small-RNA sequencing, and three biological repeats were performed.

The grafting experiment was performed at the National Citrus Breeding Center, Huazhong Agricultural University, Wuhan, China. Healthy and uniform annual branches were selected from one adult sweet orange tree as scions, and biennial seed-germinated *P. trifoliata* and sweet orange seedlings with uniform growth were selected as rootstocks. Two grafting combinations were constructed: one with sweet orange (SWO/SWO) grafted as rootstock and one with *P. trifoliata* (SWO/Pt) grafted as rootstock. Each grafting combination was used for at least eight plants. All seedlings were put into the same greenhouse with the same water, fertilizer, and pest management conditions. Five months after grafting, gene expression and DNA methylation analyses were carried out on the 5th–8th leaves above the graft union. Every three grafted seedlings were combined as one biological repeat. Two biological repeats were performed for bisulfite sequencing and small-RNA sequencing.

### Genome assembly and annotation

Illumina reads were used to estimate the *P. trifoliata* genome features by GCE software^57^. The genome size was 335 Mb, and the heterozygosity was 1.02% based on the k-mer depth distribution. Approximately 20-kb SMRT libraries were prepared according to the released protocol for the PacBio sequel platform. This generated a total sequence length of 30.49 Gb. We used Falcon/Falcon_unzip^39^ to assemble these SMRT sequences. Subsequently, the draft-assembled contigs were polished with Quiver. Finally, Pilon v.1.1.8^58^ was utilized to perform the second round of error correction with Illumina reads. The genetic map with 1934 markers was used for anchoring the assembled contigs^41^. For TE annotation, a de novo repeat library was first constructed by RepeatModeler v.1.0.11^59^ (http://www.repeatmasker.org). Then, the library was integrated with the repBase^60^ plant repeat database. Finally, RepeatMasker v.4.0.7^61^ was used to mask repeat elements.

Ab initio gene predictions, homology searches and RNA-seq analysis were integrated to predict gene models. Ab initio gene prediction and annotation were performed by Augustus v.3.2.2^62^ and GlimmerHMM v.3.0.4^63^. Then, the gene structure was further confirmed based on the published proteins and expressed sequence tags (ESTs) of citrus species by using the AAT package^64^ and Exonerate v.2.2.0^65^. For RNA-seq analysis, transcriptomes from shoot, leaf, and flower material were aligned to the genome by TopHat2 v.2.1.1^66^. Then, these sequences were subjected to genome-guided and de novo assembly by Trinity v.2.3.2^67^. The assemblies were further refined by using PASA^68^. All the predicted gene structures above were integrated by EVM^69^. Finally, the gene models were generated after annotating the UTR and alternative splicing isoforms using PASA pipeline v.2.3.3^68^. For functional annotation of protein-coding genes, nucleotide sequences of high-confidence genes were searched against the SwissProt and TrEMBL databases. The motifs and domains within gene models were identified by InterProScan v.5.32.71^70^.

### Phylogenetic tree construction

An all-vs.-all BLASTP search was first performed using the corresponding protein sequences of *P. trifoliata* and seven other citrus species. Then, gene family clustering was conducted by OrthoMCL v.2.0.9^71^. The single-copy orthologous genes were retrieved from the eight Citrinae species and aligned by Muscle v.3.8.425^72^. The poorly aligned sequences were eliminated using Gblocks^73^ with default parameters. RAxML v.8.2.12^74^ was finally used to construct the maximum-likelihood phylogenetic tree with 1000 bootstraps.

### Whole-genome bisulfite analysis

High-quality whole-genome bisulfite reads were mapped to the assembled *P. trifoliata* genome using Bismark v.0.18.1^75^. The unique mapped reads were used to identify differentially methylated cytosines and regions using the methylKit^76^ package. Bases that had coverage below 4× and had more than the 99.9th percentile of coverage were discarded. For each treatment, methylation call files corresponding to the three methylation sequence contexts were generated. The methylation levels of annotated features, including genes, promoter regions (2 kb upstream of transcription start site), and TEs, were calculated by a customized Perl script. The DMRs were identified by MethylKit package^76^. Hyper-DMR and hypo-DMR in the CG, CHG, and CHH contexts were identified using a 1000-bp window. Regions with a minimum methylation difference of 25% or for which the fold change in the methylation level was ≤0.5 and ≥2 were regarded as DMRs, and regions containing <4 methylated cytosines were removed. The DMRs were allocated to gene bodies and promoter regions. KEGG (http://www.genome.jp/kegg/) was used to understand the pathway enrichment of DMR-related genes^77^. In addition, the conversion rate of WGBS was assessed by using lambda phage DNA samples, and the conversion rate ranged from 99.54% to 99.61%.

### RNA extraction and transcriptome analysis

Total RNA from different tissues was extracted using an RNA extraction kit (RNAiso Plus, TaKaRa) following the manufacturer’s instructions. RNA-seq libraries were constructed and sequenced on the Illumina Genome Analyzer platform. The clean RNA-seq reads were mapped to the reference genome by Hisat2 v.2.0.4^78^. The correlation coefficients of biological replicates were calculated by the cor function in R. The Ballgown package^79^ was utilized to estimate gene expression levels. The Cuffdiff v.2.2.1^80^ procedure was followed to identify differentially expressed genes (FDR < 0.05). DEGs were assigned to GO terms, and GO enrichment was performed by the agriGO database^81^.

### Small RNA-seq and data analysis

sRNA sequencing raw reads were filtered first by removing the low-quality reads, filtering the contaminants, and trimming the adaptor sequences. The filtered reads were then mapped to the reference genome by Bowtie2 v.2.1.0^82^ with no mismatch. For the mapped reads, only those of 20–24 nt with counts ≥2 were retained. Then, the retained reads were further filtered for tRNAs, rRNAs, snRNAs, and snoRNAs based on their alignment to the Rfam database^83^. The remaining small RNAs were further processed to assess their positions in the chromosome and their overlap with methylated regions. For comparison of expression levels, the sRNA read counts were normalized to RPM.

### Girdling experiments

One year after the graft union completely healed, both grafting combinations (SWO/SWO and SWO/Pt) were used for the girdling experiments. The removed bark was located 3–5 cm above the graft union, and a 5-mm-wide section of bark was removed down to the xylem. Leaf samples were collected from the scion (above the girdling position) before and 10 days after girdling. Leaves from three grafted plants were combined as one biological repeat, and three biological repeats were prepared for each graft combination.

### Quantitative PCR analysis

Total RNA from all tissues was extracted using TRIzol reagent (Takara). cDNA was synthesized using 1 μg of total RNA and HiScript II QRT SuperMix for qPCR (Vazyme, R223-01). qRT-PCR was performed on an LC480 instrument (Roche) using SYBR Green PCR Mastermix according to the manufacturer’s instructions (Kapa, RR420). The cycling conditions included incubation for 5 min at 95 °C followed by 40 cycles of amplification (95 °C for 5 s and 60 °C for 35 s). Using the citrus β-actin gene as the internal reference gene, relative gene expression values were calculated using the 2^−ΔΔCt^ method^84^. miRNA expression was detected by stem-loop qRT-PCR^85^. Three independent biological replicates and at least three technical replicates were performed. All the primers used in qRT-PCR are listed in Supplementary Table 17.

### Data access

The assembled genome sequences of *P. trifoliata* have been deposited at DDBJ/ENA/GenBank under accession number VKKW00000000 and can also be downloaded at our website http://citrus.hzau.edu.cn/orange/download/index.php. Whole-genome sequencing data, transcriptome data, whole-genome bisulfite sequencing data, and sRNA data have also been deposited in the NCBI database, and the accession numbers of each sample are recorded in Supplementary Tables 10, 11,14, 15, 18, and 19.

## Supplementary information

Supplementary Figures

Supplementary Tables

Supplementary 8,9,12,13 and 16

## References

[1] Melnyk CW, Meyerowitz EM (2015). Plant grafting. Curr. Biol..

[2] He W (2018). Dissection of the mechanism for compatible and incompatible graft combinations of *Citrus grandis* (L.) Osbeck (‘Hongmian Miyou’). Int. J. Mol. Sci..

[3] Tietel Z (2020). Impact of scion/rootstock reciprocal effects on metabolomics of fruit juice and phloem sap in grafted *Citrus reticulata*. PLoS ONE.

[4] Yang Y (2017). Differential expression analysis of genes related to graft union healing in *Pyrus ussuriensis* Maxim by cDNA-AFLP. Sci. Hortic..

[5] Hudina M, Orazem P, Jakopic J, Stampar F (2014). The phenolic content and its involvement in the graft incompatibility process of various pear rootstocks (*Pyrus communis* L.). J. Plant Physiol..

[6] Assuncao M, Santos C, Brazao J, Eiras-Dias JE, Fevereiro P (2019). Understanding the molecular mechanisms underlying graft success in grapevine. BMC Plant Biol..

[7] Gakpetor PM, Mohammed H, Moreti D, Nassar NMA (2017). Periclinal chimera technique: new plant breeding approach. Genet. Mol. Res..

[8] Robert ML, Juarez-Gomez J, Chaires-Pacheco M, Pena-Ramirez YJ (2019). Successive grafting confers juvenility traits to adult Spanish red cedar (*Cedrela odorata* Linnaeus): a tool for the rescue of selected materials. N. For..

[9] Mudge K, Janick J, Scofield S, Goldschmidt EE (2009). A history of grafting. Hortic. Rev..

[10] Zhang L, Marguerit E, Rossdeutsch L, Ollat N, Gambetta GA (2016). The influence of grapevine rootstocks on scion growth and drought resistance. Theor. Exp. Plant Physiol..

[11] Huang Y, Tang R, Cao Q, Bie Z (2009). Improving the fruit yield and quality of cucumber by grafting onto the salt tolerant rootstock under NaCl stress. Sci. Hortic..

[12] Rouphael Y, Schwarz D, Krumbein A, Colla G (2010). Impact of grafting on product quality of fruit vegetables. Sci. Hortic..

[13] Tramontini S, Vitali M, Centioni L, Schubert A, Lovisolo C (2013). Rootstock control of scion response to water stress in grapevine. Environ. Exp. Bot..

[14] Agut B, Gamir J, Jaques JA, Flors V (2016). Systemic resistance in citrus to *Tetranychus urticae* induced by conspecifics is transmitted by grafting and mediated by mobile amino acids. J. Exp. Bot..

[15] Kumar P, Rouphael Y, Cardarelli M, Colla G (2017). Vegetable grafting as a tool to improve drought resistance and water use efficiency. Front. Plant Sci..

[16] Wang J, Jiang L, Wu R (2017). Plant grafting: how genetic exchange promotes vascular reconnection. N. Phytol..

[17] Uthup TK, Karumamkandathil R, Ravindran M, Saha T (2018). Heterografting induced DNA methylation polymorphisms in *Hevea brasiliensis*. Planta.

[18] Wu R (2013). Inter-species grafting caused extensive and heritable alterations of DNA methylation in *Solanaceae* plants. PLoS ONE.

[19] Avramidou E (2015). Global DNA methylation changes in Cucurbitaceae inter-species grafting. Crop Breed. Appl. Biot..

[20] Cao L (2016). Heritability and reversibility of DNA methylation induced by in vitro grafting between *Brassica juncea* and *B. oleracea*. Sci. Rep..

[21] Yu N (2018). Maintenance of grafting-induced epigenetic variations in the asexual progeny of *Brassica oleracea* and *B. juncea* chimera. Plant J..

[22] Goldschmidt EE (2014). Plant grafting: new mechanisms, evolutionary implications. Front. Plant Sci..

[23] Castle WS (2010). A career perspective on citrus rootstocks, their development, and commercialization. Hortic. Sci..

[24] Stover E, Inch S, Richardson ML, Hall DG (2016). Conventional citrus of some scion/ rootstock combinations show field tolerance under high huanglongbing disease pressure. Hortic. Sci..

[25] Benjamin G, Tietel Z, Porat R (2013). Effects of rootstock/scion combinations on the flavor of citrus fruit. J. Agric. Food Chem..

[26] Laino P (2016). Rootstock–scion interaction affecting citrus response to CTV infection: a proteomic view. Physiol. Plant..

[27] Souza JD (2017). Different adaptation strategies of two citrus scion/rootstock combinations in response to drought stress. PLoS ONE.

[28] Saini MK, Capalash N, Kaur C, Singh SP (2019). Comprehensive metabolic profiling to decipher the influence of rootstocks on fruit juice metabolome of Kinnow (*C. nobilis* × *C. deliciosa*). Sci. Horticul..

[29] Albrecht U, Tripathi I, Bowman KD (2019). Rootstock influences the metabolic response to *Candidatus Liberibacter asiaticus* in grafted sweet orange trees. Trees.

[30] Castle W. S., Tucker, D. P. H., Krezdom, A. H. & Youtsey, C. O. Rootstocks for Florida citrus. Institute of Food and Agriculture Science/University of Florida. 1-22 (1989).

[31] Rom R. C. & Carlson, R. F. *Rootstocks for Fruit Crops*. 384–385 (A Wiley-Interscience Publication, 1987).

[32] Ghobakhloo M, Dizadji A, Yamchi A (2019). A real-time PCR assay for detection and absolute quantitation of *Citrus exocortis* viroid in two sensitive and tolerant rootstocks. Crop Prot..

[33] Al-Jaleel A, Zekri M, Hammam Y (2005). Yield, fruit quality, and tree health of ‘Allen Eureka’ lemon on seven rootstocks in Saudi Arabia. Sci. Hortic..

[34] Befu M, Kitajima A, Ling YX, Hasegawa K (2008). Classification of ‘Tosa-Buntan’ pummelo (*Citrus grandis* [L.] Osb.), ‘Washington’ navel orange (*C. sinensis* [L.] Osb.) and trifoliate orange (*Poncirus trifoliata* [L.] Raf.) chromosomes using young leaves. J. Jpn. Soc. Hortic. Sci..

[35] Gong XQ, Liu JH (2012). Genetic transformation and genes for resistance to abiotic and biotic stresses in *Citrus* and its related genera. Plant Cell Tissue Org..

[36] Boava LP (2011). Global gene expression of *Poncirus trifoliata*, Citrus sunki and their hybrids under infection of *Phytophthora parasitica*. BMC Genom..

[37] George J, Lapointe S (2018). Host plant resistance associated with *Poncirus trifoliata* influence oviposition, development and adult emergence of *Diaphorina citri* (Hemiptera: Liviidae). Pest Manag. Sci..

[38] Hu, Y. Studies on the Genetic Diversity of Trifoliate Orange (*Poncirus trifoliata* [L.] Raf) and Genetics of its Hybrids with Red Tangerine (*Citrus reticulata* Blanco). MD dissertation, Huazhong Agricultural University (2015).

[39] Chin CS (2016). Phased diploid genome assembly with single-molecule real-time sequencing. Nat. Methods.

[40] Simao FA, Waterhouse RM, Ioannidis P, Kriventseva EV, Zdobnov EM (2015). BUSCO: assessing genome assembly and annotation completeness with single-copy orthologs. Bioinformatics.

[41] Zheng X (2019). SLAF-based construction of a high-density genetic map and its application in QTL mapping of carotenoids content in citrus fruit. J. Agric. Food Chem..

[42] Wu GA (2014). Sequencing of diverse mandarin, pummelo and orange genomes reveals complex history of admixture during citrus domestication. Nat. Biotechnol..

[43] Xu Q (2013). The draft genome of sweet orange (*Citrus sinensis*). Nat. Genet..

[44] Wang X (2017). Genomic analyses of primitive, wild and cultivated citrus provide insights into asexual reproduction. Nat. Genet..

[45] Wang L (2018). Genome of wild mandarin and domestication history of mandarin. Mol. Plant.

[46] Grosser JW, Gmitter FGJ, Chandler JL (1998). Intergeneric somatic hybrid plants from sexually incompatible woody species: *Citrus sinensis* and *Severinia disticha*. Theor. Appl. Genet..

[47] Hussain S, Luro F, Costantino G, Ollitrault P, Morillon R (2012). Physiological analysis of salt stress behaviour of citrus species and genera: low chloride accumulation as an indicator of salt tolerance. S. Afr. J. Bot..

[48] Xu J, Xu H, Xu Q, Deng X (2014). Characterization of dna methylation variations during fruit development and ripening of sweet orange. Plant Mol. Biol. Rep..

[49] Matzke MA, Mosher RA, RNA-directed DNA (2014). methylation: an epigenetic pathway of increasing complexity. Nat. Rev. Genet..

[50] Saunt J (1990). Citrus Varieties of the World..

[51] Yelenosky G (1985). Cold hardiness in citrus. Hortic. Rev..

[52] Peng, Z. et al. A chromosome-scale reference genome of trifoliate orange (*Poncirus trifoliata*) provides insights into disease resistance, cold tolerance and genome evolution in *Citrus*. *Plant J*. 10.1111/tpj.14993 (2020). 10.1111/tpj.14993 PMC7756384 32985030

[53] Koepke T, Dhingra A (2013). Rootstock scion somatogenetic interactions in perennial composite plants. Plant Cell Rep..

[54] Pant BD, Buhtz A, Kehr J, Scheible WR (2008). MicroRNA399 is a long-distance signal for the regulation of plant phosphate homeostasis. Plant J..

[55] Molnar Attila (2010). Small silencing RNAs in plants are mobile and direct epigenetic modification in recipient cells. Science.

[56] Lewsey MG (2016). Mobile small RNAs regulate genome-wide DNA methylation. Proc. Natl Acad. Sci. USA.

[57] Liu, B. et al. Estimation of genomic characteristics by analyzing kmer frequency in de novo genome projects. Preprint at http://arxiv.org/abs/1308 (2013).

[58] Walker BJ (2014). Pilon: an integrated tool for comprehensive microbial variant detection and genome assembly improvement. PLoS ONE.

[59] Smit, A. F. A. & Hubley, R. RepeatModeler Open-1.0 http://www.repeatmasker.org (2008–2015).

[60] Bao W, Kojima KK, Kohany O (2015). Repbase Update, a database of repetitive elements in eukaryotic genomes. Mob. DNA.

[61] Smit, A. F. A., Hubley, R. & Green, P. RepeatMasker Open-4.0. http://www.repeatmasker.org (1996–2015).

[62] Stanke M, Diekhans M, Baertsch R, Haussler D (2008). Using native and syntenically mapped cDNA alignments to improve de novo gene finding. Bioinformatics.

[63] Majoros WH, Pertea M, Salzberg SL (2004). TigrScan and GlimmerHMM: two open source ab initio eukaryotic gene-finders. Bioinformatics.

[64] Huang X, Adams MD, Zhou H, Kerlavage AR (1997). A tool for analyzing and annotating genomic sequences. Genomics.

[65] Slater GS, Birney E (2005). Automated generation of heuristics for biological sequence comparison. BMC Bioinforma..

[66] Kim D (2013). TopHat2: accurate alignment of transcriptomes in the presence of insertions, deletions and gene fusions. Genome Biol..

[67] Grabherr MG (2011). Full-length transcriptome assembly from RNA-Seq data without a reference genome. Nat. Biotech..

[68] Haas BJ (2003). Improving the Arabidopsis genome annotation using maximal transcript alignment assemblies. Nucleic Acids Res..

[69] Haas BJ (2008). Automated eukaryotic gene structure annotation using EVidenceModeler and the Program to Assemble Spliced Alignments. Genome Biol..

[70] Jones P (2014). InterProScan 5: genome-scale protein function classification. Bioinformatics.

[71] Li L, Stoeckert CJ, Roos DS (2003). OrthoMCL: identification of ortholog groups for eukaryotic genomes. Genome Res..

[72] Edgar RC (2004). MUSCLE: multiple sequence alignment with high accuracy and high throughput. Nucleic Acids Res..

[73] Talavera G, Castresana J (2007). Improvement of phylogenies after removing divergent and ambiguously aligned blocks from protein sequence alignments. Syst. Biol..

[74] Stamatakis A (2014). RAxML version 8: a tool for phylogenetic analysis and post-analysis of large phylogenies. Bioinformatics.

[75] Krueger F, Andrews SR (2011). Bismark: a flexible aligner and methylation caller for Bisulfite-Seq applications. Bioinformatics.

[76] Akalin A (2012). methylKit: a comprehensive R package for the analysis of genome-wide DNA methylation profiles. Genome Biol..

[77] Kanehisa M (2008). KEGG for linking genomes to life and the environment. Nucleic Acids Res..

[78] Kim D, Langmead B, Salzberg SL (2015). HISAT: a fast spliced aligner with low memory requirements. Nat. Methods.

[79] Pertea M, Kim D, Pertea GM, Leek JT, Salzberg SL (2016). Transcript-level expression analysis of RNA-seq experiments with HISAT, StringTie and Ballgown. Nat. Protoc..

[80] Trapnell C (2010). Transcript assembly and quantification by RNA-Seq reveals unannotated transcripts and isoform switching during cell differentiation. Nat. Biotech..

[81] Tian T (2017). agriGO v2.0: a GO analysis toolkit for the agricultural community, 2017 update. Nucleic Acids Res..

[82] Langmead B, Salzberg SL (2012). Fast gapped-read alignment with Bowtie 2. Nat. Methods.

[83] Griffiths-Jones S (2005). Rfam: annotating non-coding RNAs in complete genomes. Nucleic Acids Res..

[84] Livak KJ, Schmittgen TD (2001). Analysis of relative gene expression data using real-time quantitative PCR and the 2^−ΔΔCT^ method. Methods.

[85] Varkonyi-Gasic E, Wu R, Wood M, Walton EF, Hellens RP (2007). Protocol: a highly sensitive RT-PCR method for detection and quantification of microRNAs. Plant Methods.

