# Supplementary material for: Genome of a citrus rootstock and global DNA demethylation caused by heterografting
Source: Hortic Res. 2021 Apr 1;8:69. doi: 10.1038/s41438-021-00505-2 (PMC8012640; doi:10.1038/s41438-021-00505-2)
Supplement: Supplementary file 1 — Supplementary Figures [file 41438_2021_505_MOESM1_ESM.doc]

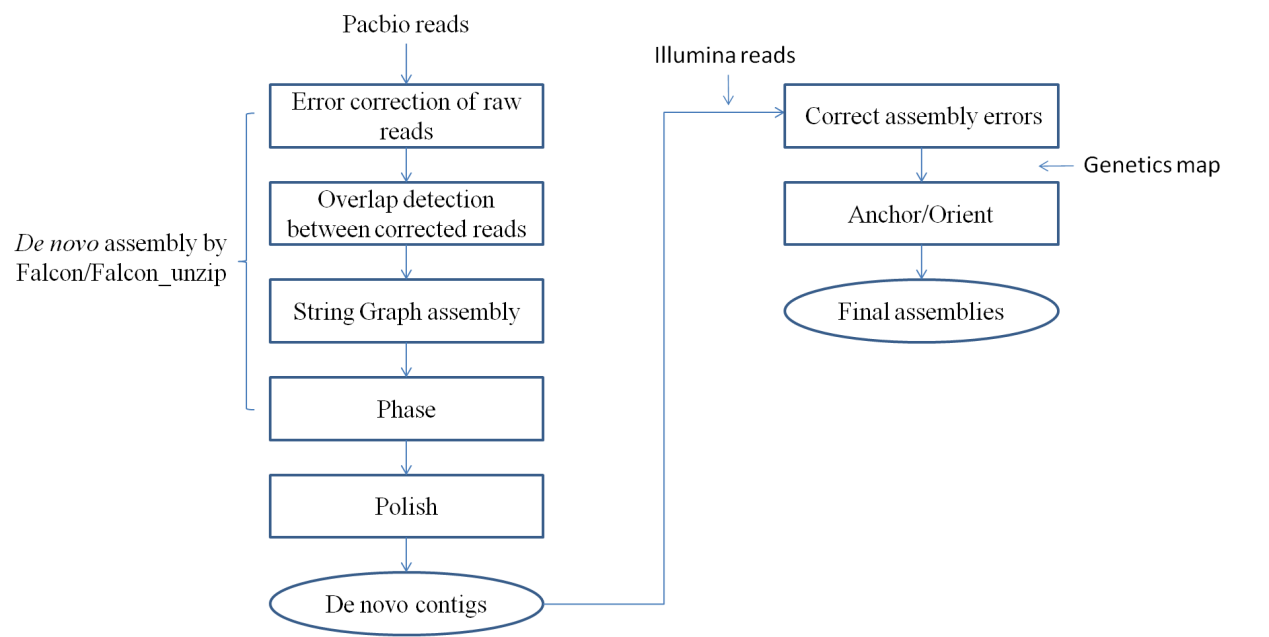


**Supplementary Figure 1.** The pipeline of genome assembly for *Poncirus trifoliate.*


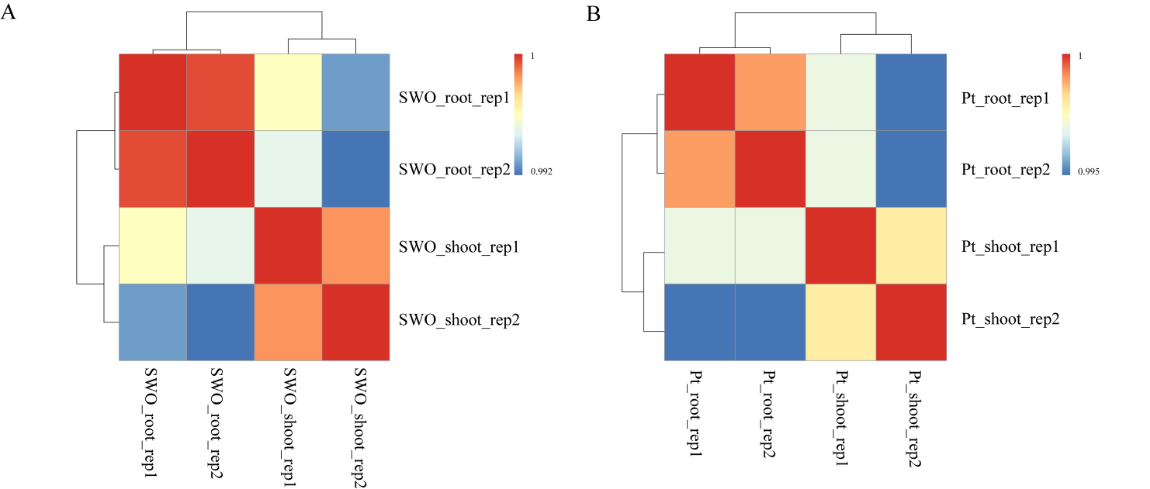


**Supplementary Figure 2.** Correlation and hierarchical clustering of WGBS from shoot and root of P.trifoliata (A) and sweet orange (B). Heat map shows the correlation among the shoot and root and the repeatability between biological replicates. A high degree of correlation is indicated by dark red color.


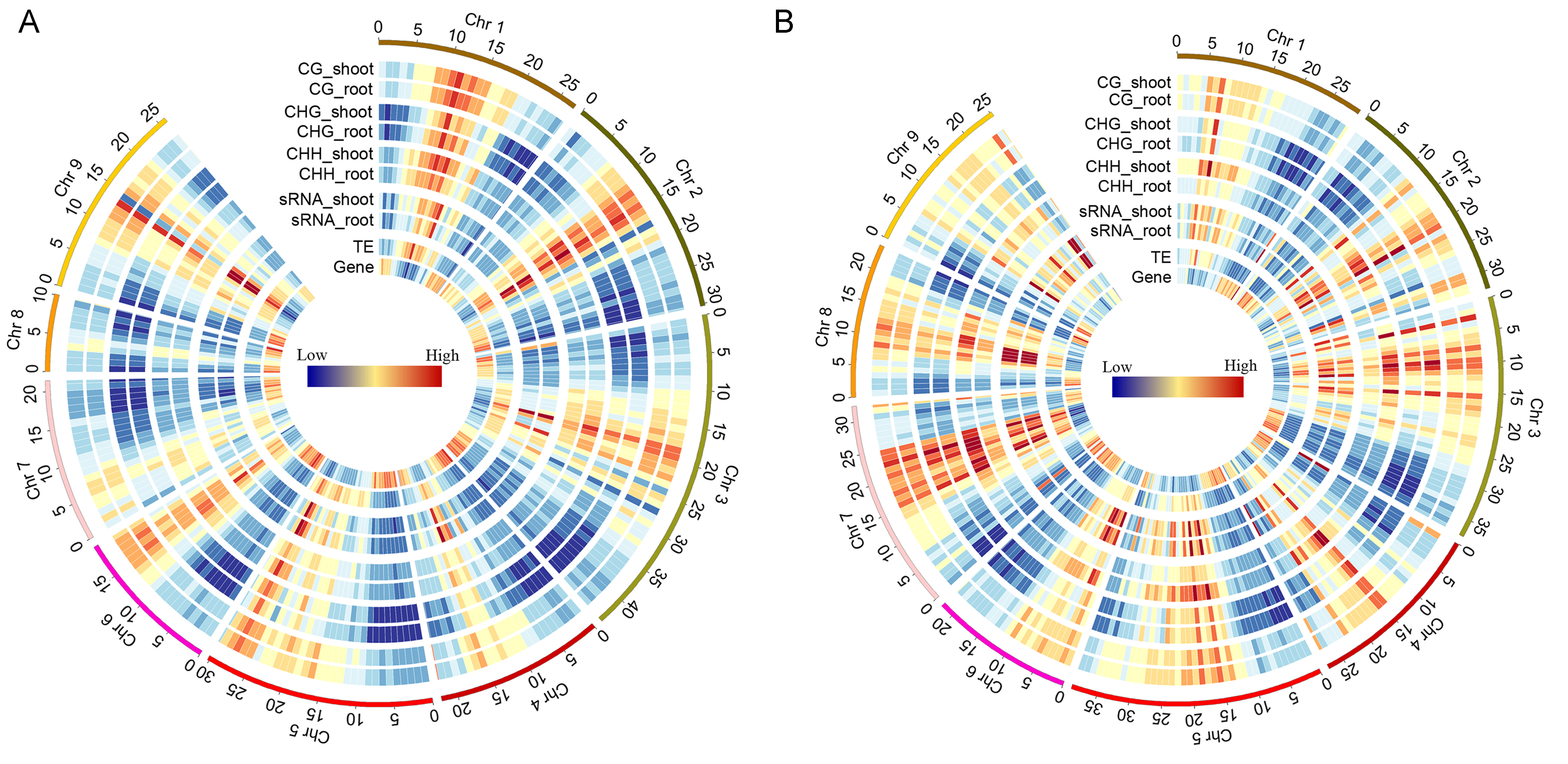


**Supplementary Figure 3.** DNA methylation landscape of *P. trifoliata* (A) and sweet orange (B).


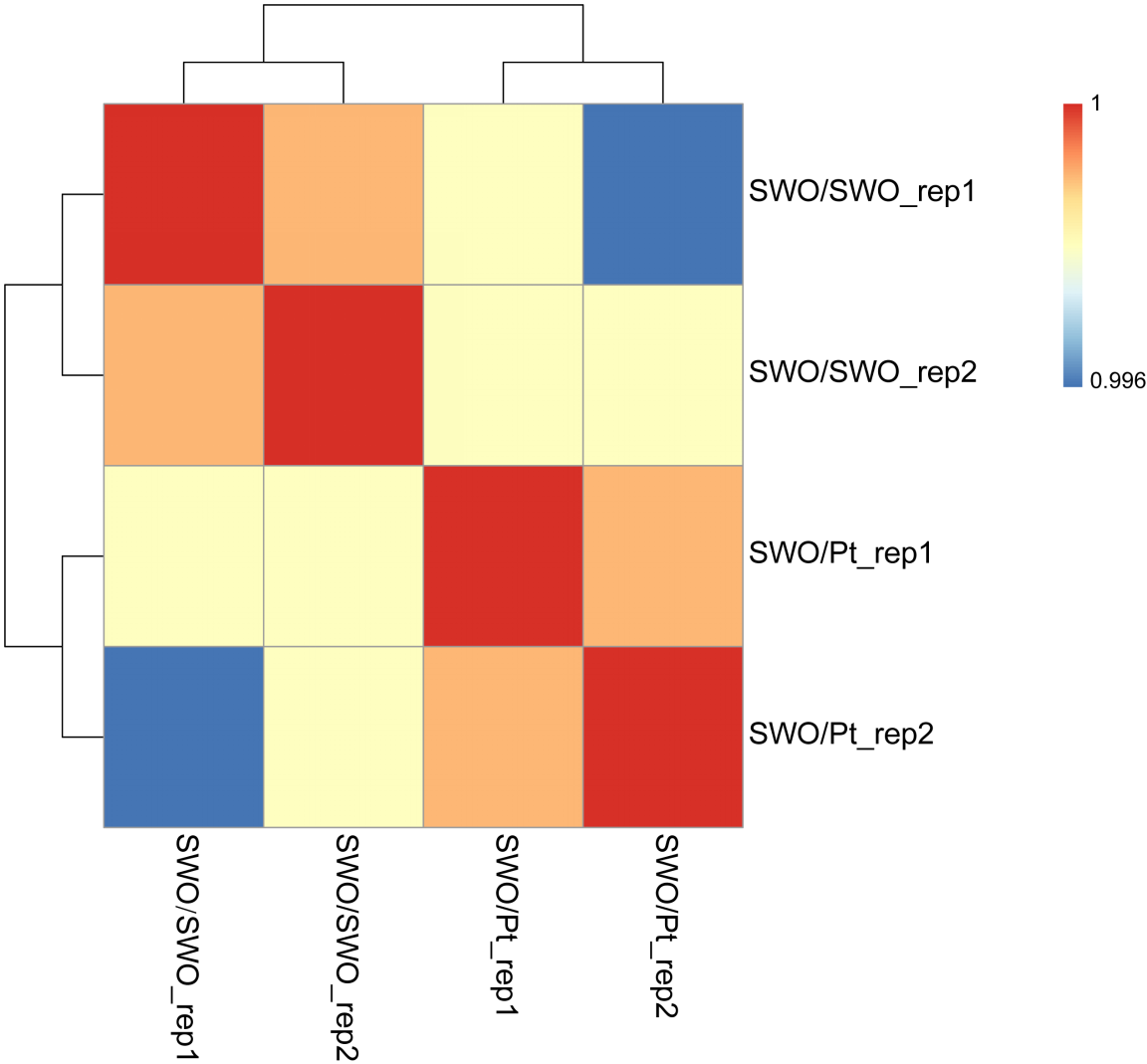


**Supplementary Figure 4**. Correlation and hierarchical clustering of WGBS from leaves of scion from SWO/SWO and SWO/Pt. Heat map shows the correlation among the shoot and root and the repeatability between biological replicates. A high degree of correlation is indicated by dark red color.


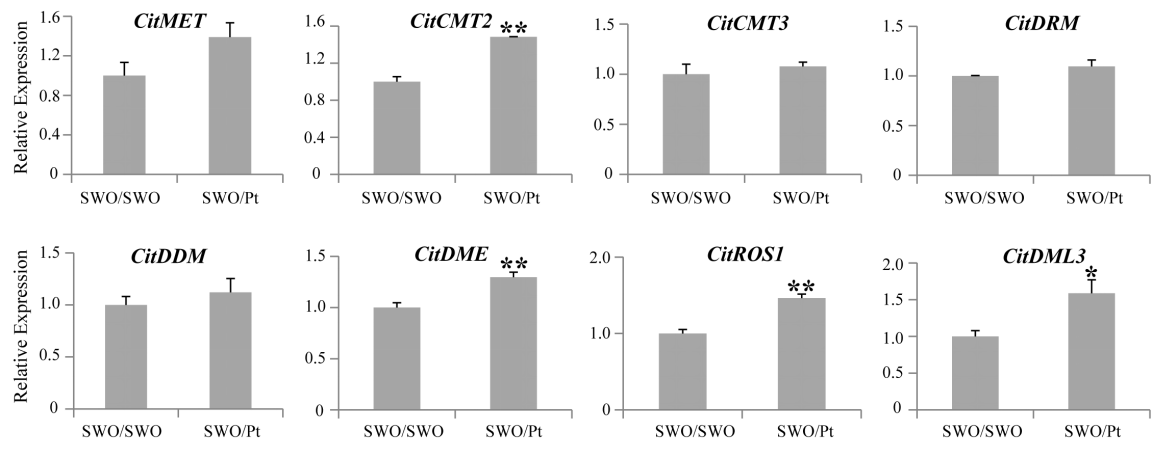


**Supplementary Figure 5.** The expression of DNA methylase genes and demethylase genes. Data are mean ± s.d.; n = 3 technical replicates of 2 pooled tissue samples. * and ** indicate significantly difference at *P* < 0.05 and *P* < 0.01, respectively.


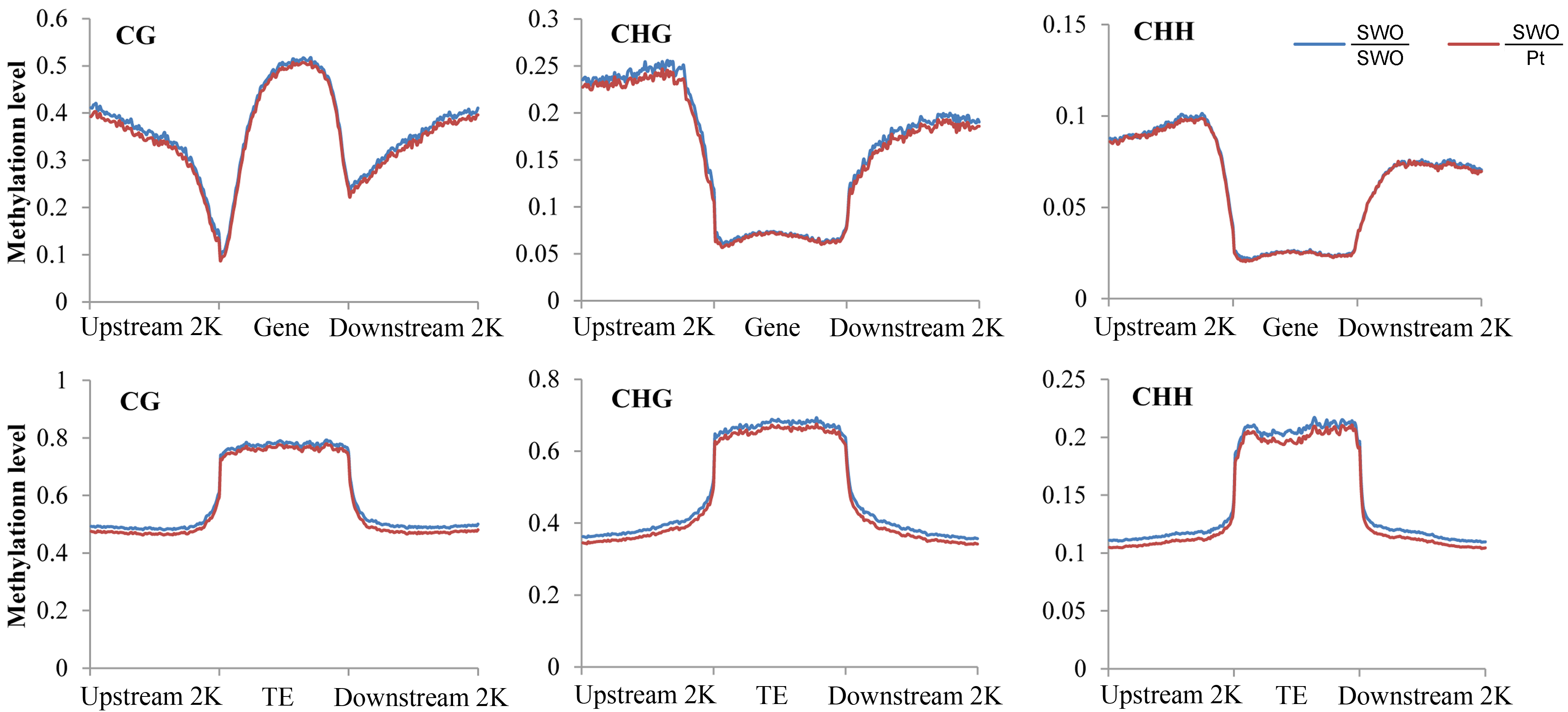


**Supplementary Figure 6.**The DNA methylation patterns in gene and TE regions in scions of hetero-grafting and auto-grafting plants.


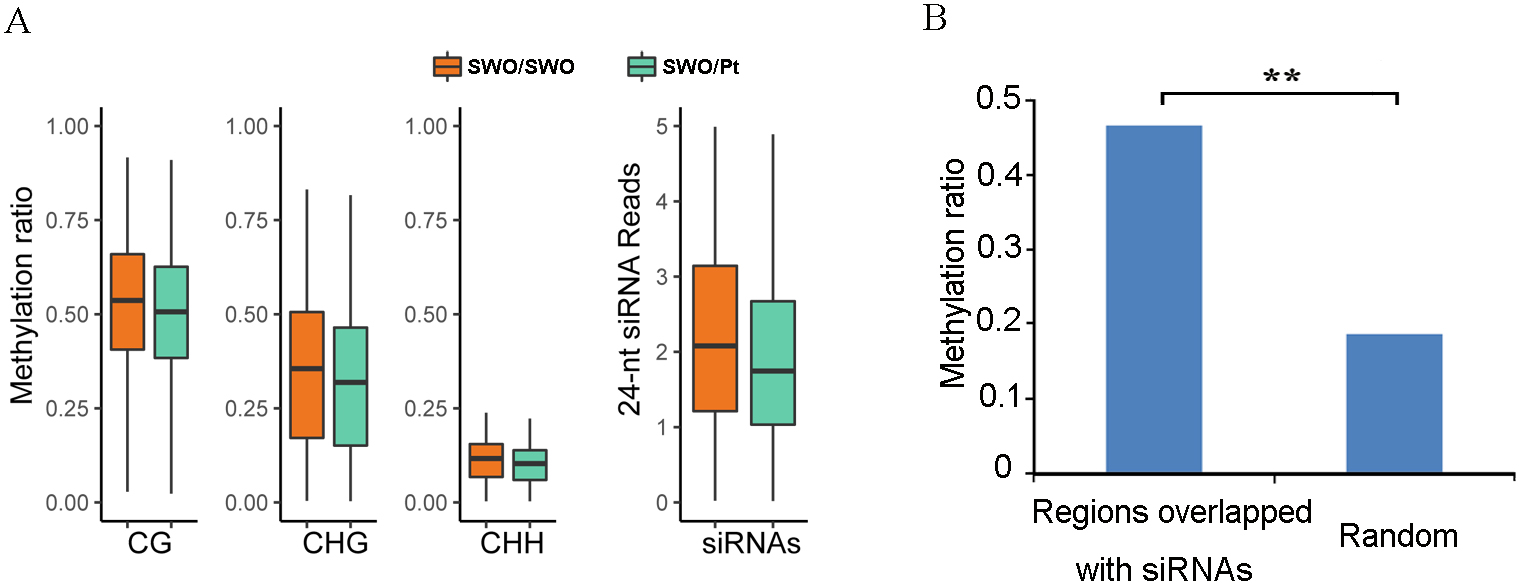


**Supplementary Figure 7.** Association between grafting-induced siRNA decrease and DNA hypomethylation. (A) Comparisons of methylation levels and 24-nt siRNAs between SWO/SWO and SWO/Pt. For the calculation of methylation and siRNA levels, chromosomes were divided into 1 Mb windows that slide 200 kb. (B) DMRs are significantly associated with siRNA clusters as compared to random genomic regions (**P value <0.01, as determined using Fisher’s exact test).


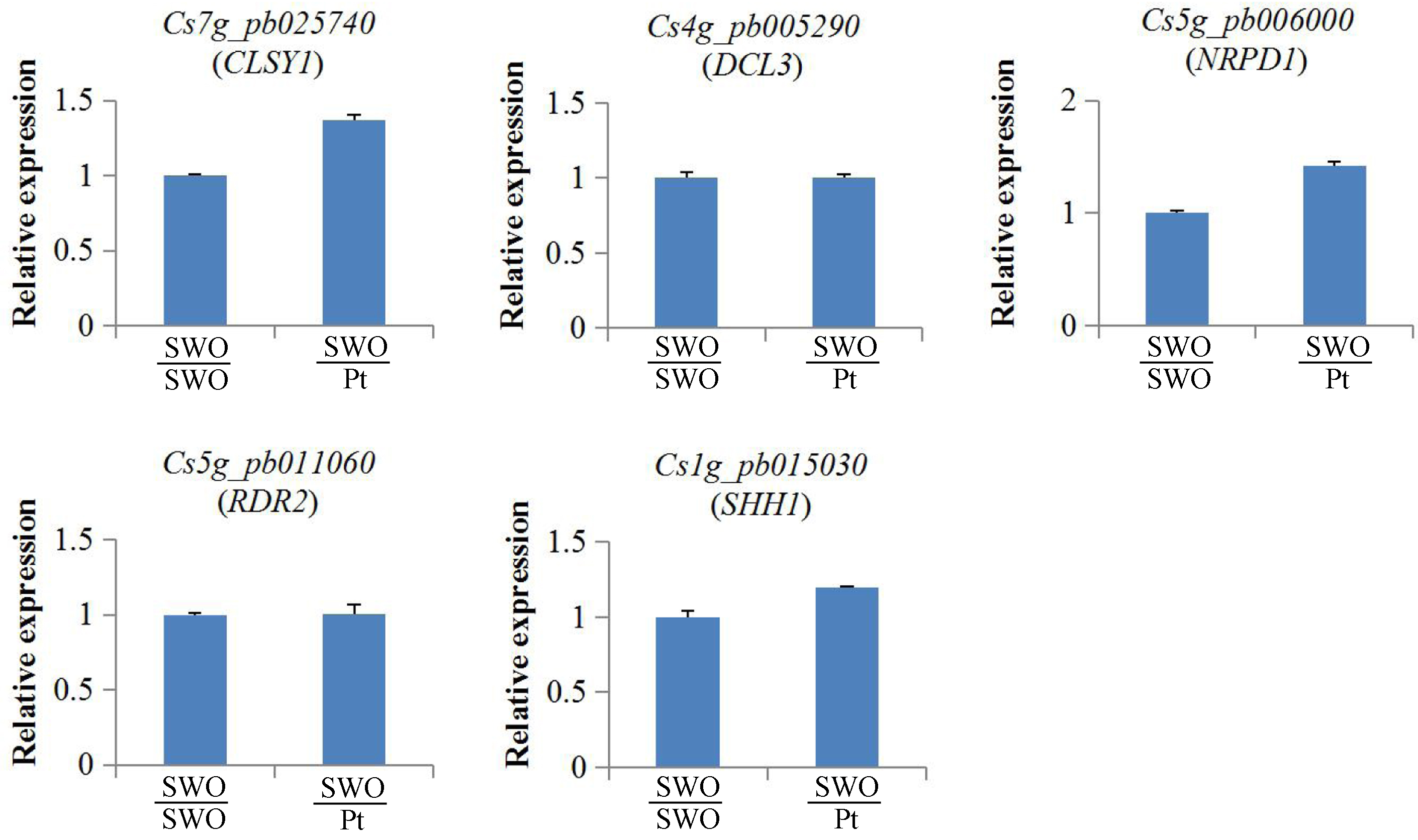


**Supplementary Figure 8.** qRT-PCR validation of the key genes involved in 24-nt siRNA biogenesis in scion of SWO/SWO and SWO/Pt. Data are mean ± s.d.; n = 3 technical replicates of 2 pooled tissue samples.
