# Supplementary material for: Genome of a citrus rootstock and global DNA demethylation caused by heterografting
Source: Hortic Res. 2021 Apr 1;8:69. doi: 10.1038/s41438-021-00505-2 (PMC8012640; doi:10.1038/s41438-021-00505-2)
Supplement: Supplementary file 2 — Supplementary Tables [file 41438_2021_505_MOESM2_ESM.doc]

| **Supplementary Table 1. Information of the 16 SSR markers used for heterozygosity analysis of the 169 *Poncirus trifoliata* accessions.** | | |
| --- | --- | --- |
| **Name** | **Forward primer (5'-3')** | **Reverse primer (5'-3')** |
| Ma3-153 | CTGTTGCTGCTCTTGGATCA | GTTCCGGATTGAACCATGTC |
| Ma3-73 | ATCCTCATTTGTTTTCCCCC | GTTGGCTTGTGATCGGAGTT |
| Ma4-178 | TTTTTCTTTCCCCCTCATCA | ATAACTCCCCGACTGCCTCT |
| Ma3-143 | AATTTGTTGCTGTGCTTCCC | GATCTGGGTTGGATCCTTGA |
| Ma6-29 | CACTTCTAAACCCGAACCCA | TTGGAGGAATCAAGAGGGTG |
| Ma2-1251 | TTTATTATCTCTGTTCTGGTCCG | TTGCCTCTTTCCACCGTTAG |
| CAT01 | GCTTTCGATCCCTCCACATA | GATCCCTACAATCCTTGGTCC |
| HC4-9 | CTGGCCTCATTTCTCGTTTC | ATTTGATGGGACCTTCCTCC |
| Ma4-32 | CGCGAAATTGAACCATTTTT | CTAAATGCCCAAATCCCAGA |
| Ma4-116 | CTTCTTCTGGCCATCAAAGC | GCATGATTTACATTCTCGTCCC |
| Ma4-129 | GTCCGTTCTCCTCGCTCTTC | TGTAGGTAGGCAACGGAAGG |
| P29 | CAGTGCTGGCATTACAAACG | AGGCAAGAATTGTTGGTTGG |
| P58 | TTTTTATGCGGGACGTTAGTT | GAGCTCTGCCATCAAACCTG |
| TAA1 | GACAACATCAACAACAGCAAGAGC | AAGAAGAAGAGCCCCCATTAGC |
| TAA15 | GAAAGGGTTACTTGACCAGGC | CTTCCCAGCTGCACAAGC |
| TAA41 | AGGTCTACATTGGCATTGTC | ACATGCAGTGCTATAATGAATG |

**Supplementary Table 2. PacBio sequencing data for assembly of *P. trifoliata*.**

| **Library** | **Insert size(Kb)** | **Total data(Gb)** | **Sequence coverage** | **Bioproject** |
| --- | --- | --- | --- | --- |
| ZK8 | 20 | 30.49 | 91.01X | PRJNA554539 |

**Supplementary Table 3. Illumina sequencing data for assembly of *P. trifoliata*.**

| **Library** | **Number of  reads pairs** | **Read length  (bp)** | **Total Bases (bp)** | **Mapping rates** | **GC content** | **Bioproject** |
| --- | --- | --- | --- | --- | --- | --- |
| ZK8 | 48666419 | 150 | 14,599,925,700 | 96.6% | 37% | PRJNA554539 |

**Supplementary Table 4. Details of the anchoring of contigs on the *P. trifoliata* linkage map.**

| **Linkage groups** | **Total markers** | **Sequence known markers** | **Anchored markers** | **Number of contigs** | **Length** |
| --- | --- | --- | --- | --- | --- |
| LG1 | 235 | 235 | 233 | 34 | 27,262,294 |
| LG2 | 221 | 221 | 219 | 26 | 30,287,271 |
| LG3 | 412 | 412 | 211 | 48 | 43,675,693 |
| LG4 | 199 | 199 | 197 | 22 | 22,163,939 |
| LG5 | 243 | 243 | 238 | 35 | 30,279,337 |
| LG6 | 145 | 145 | 143 | 16 | 19,618,469 |
| LG7 | 230 | 230 | 228 | 26 | 21,475,025 |
| LG8 | 67 | 67 | 66 | 4 | 10,129,192 |
| LG9 | 182 | 182 | 180 | 20 | 26,029,262 |
| Total | 1934 | 1934 | 1715 | 231 | 230,920,482 |

**Supplementary Table 5. Summary of transposable elements in *P. trifoliata*.**

| **TE classification** | **Copies(number)** | **Length(bp)** | **Percent of genome** |
| --- | --- | --- | --- |
| LTR/Gyspy | 29186 | 29277995 | 9.66% |
| LTR/Copia | 33072 | 28593813 | 9.43% |
| LTR/Caulimovirus | 10376 | 14507207 | 4.79% |
| LINE | 6777 | 5011401 | 1.65% |
| SINE | 624 | 126664 | 0.04% |
| DNA/hat | 17582 | 6389709 | 2.11% |
| DNA/EnSpm | 71 | 35925 | 0.01% |
| DNA/PIF | 6335 | 2208976 | 0.73% |
| DNA/MuDR | 8951 | 4172503 | 1.38% |
| DNA/Tc1 | 5516 | 2000515 | 0.66% |
| DNA/Helitron | 3487 | 1805186 | 0.60% |
| DNA/CMC | 6052 | 2424017 | 0.80% |
| Simple_repeat | 91190 | 4357457 | 1.44% |
| Low_complexity | 17781 | 852655 | 0.28% |
| Satellite | 948 | 127218 | 0.04% |
| Unknown | 92870 | 39015247 | 12.87% |
| Total |  | 140906488 | 46.49% |

**Supplementary Table 6. Genome statistics of *P. trifoliata*, *A.buxfoliata* and cultivated citrus.**

|  | Sequencing platform | Assembled length (Mb) | Contig N50 (Mb) | Number of gene models | TE percentage | Completeness by BUSCO |
| --- | --- | --- | --- | --- | --- | --- |
| *P. trifoliata* (This study) | Pacbio | 303 | 1.17 | 25680 | 46.5% | 97.4% |
| *P. trifoliata* (Previous study)^1^ | Pacbio | 265 | 0.84 | 25538 | 42.6% | 97.2% |
| *A. Buxfoliata^2^* | Illumina | 288 | 0.02 | 28420 | 43.92% | 95.0% |
| *C. Reticulata^3^* | Illumina | 339 | 0.02 | 28820 | 47.06% | 95.4% |
| *C. Sinensis^4^* | Illumina | 301 | 0.05 | 29445 | 46.72% | 93.3% |
| *C.medica^2^* | Illumina | 368 | 0.05 | 32579 | 45.00% | 95.0% |
| *C.grandis^2^* | Pacbio | 345 | 2.18 | 30123 | 48.67% | 95.5% |
| *C.ichangesis^2^* | Illumina | 335 | 0.08 | 32067 | 44.55% | 96.2% |

**Supplementary Table 7. Gene Ontology category enrichment for genes specific to *P. trifoliata* compared to three cultivated citrus species.**

| **GO term** | **Ontology** | **Description** | **p-value** |
| --- | --- | --- | --- |
| GO:0001659 | P | temperature homeostasis | 7.70E-05 |
| GO:0050826 | P | response to freezing | 7.70E-05 |
| GO:0009409 | P | response to cold | 7.70E-05 |
| GO:0042309 | P | homoiothermy | 7.70E-05 |
| GO:0048871 | P | multicellular organismal homeostasis | 7.70E-05 |
| GO:0009266 | P | response to temperature stimulus | 0.00061 |
| GO:0007217 | P | tachykinin receptor signaling pathway | 0.018 |
| GO:0065004 | P | protein-DNA complex assembly | 0.028 |
| GO:0071824 | P | protein-DNA complex subunit organization | 0.028 |
| GO:0006325 | P | chromatin organization | 0.032 |
| GO:0031497 | P | chromatin assembly | 0.033 |
| GO:0006334 | P | nucleosome assembly | 0.033 |
| GO:0034728 | P | nucleosome organization | 0.033 |
| GO:0006333 | P | chromatin assembly or disassembly | 0.037 |
| GO:0006323 | P | DNA packaging | 0.04 |
| GO:0007200 | P | phospholipase C-activating G-protein  coupled receptor signaling pathway | 0.043 |
| GO:0015671 | P | oxygen transport | 0.044 |
| GO:0015669 | P | gas transport | 0.044 |
| GO:0008171 | F | O-methyltransferase activity | 5.10E-07 |
| GO:0050825 | F | ice binding | 7.70E-05 |
| GO:0050824 | F | water binding | 7.70E-05 |
| GO:0031490 | F | chromatin DNA binding | 0.0037 |
| GO:0043566 | F | structure-specific DNA binding | 0.0037 |
| GO:0031492 | F | nucleosomal DNA binding | 0.0037 |
| GO:0031491 | F | nucleosome binding | 0.0043 |
| GO:0004692 | F | cGMP-dependent protein kinase activity | 0.0049 |
| GO:0004690 | F | cyclic nucleotide-dependent protein kinase activity | 0.0049 |
| GO:0036318 | F | peptide pheromone receptor activity | 0.014 |
| GO:0004933 | F | mating-type a-factor pheromone receptor activity | 0.017 |
| GO:0008502 | F | melatonin receptor activity | 0.028 |
| GO:0005184 | F | neuropeptide hormone activity | 0.032 |
| GO:0004932 | F | mating-type factor pheromone receptor activity | 0.038 |
| GO:0019825 | F | oxygen binding | 0.04 |
| GO:0016503 | F | pheromone receptor activity | 0.046 |
| GO:0044427 | C | chromosomal part | 0.011 |
| GO:0000785 | C | chromatin | 0.012 |
| GO:0000786 | C | nucleosome | 0.028 |
| GO:0032993 | C | protein-DNA complex | 0.028 |
| GO:0044815 | C | DNA packaging complex | 0.03 |

**Supplementary Table 8. Gene Ontology category enrichment for the genes highly expressed in root of *P. trifoliata*.**

In separate EXCEL file.

**Supplementary Table 9. Gene Ontology category enrichment for the genes highly expressed in root of sweet orange.**

In separate EXCEL file.

**Supplementary Table 10. Summary of the bisulfite sequencing data for *P. trifoliata* and sweet orange.**

| **Sample** | **Number of raw  reads pairs** | **Read length  (bp)** | **Total Bases  (bp)** | **Mapping rates (%)** | **SRR id** | **Conversion rate** |
| --- | --- | --- | --- | --- | --- | --- |
| Pt_shoot _rep1 | 63,541,484 | 150 | 19,062,445,200 | 76.0 | SRR9723203 | 99.61% |
| Pt_shoot _rep2 | 82,425,676 | 150 | 24,727,702,800 | 73.8 | SRR9733446 | 99.58% |
| Pt_root _rep1 | 81,041,754 | 150 | 24,312,526,200 | 60.3 | SRR9733865 | 99.57% |
| Pt_root_rep2 | 60,357,700 | 150 | 18,107,310,000 | 60.4 | SRR9733873 | 99.56% |
| SWO_shoot_rep1 | 71,753,314 | 150 | 21,525,994,200 | 71.2 | SRR9793824 | 99.60% |
| SWO_shoot_rep2 | 49,597,717 | 150 | 14,879,315,100 | 69.4 | SRR9821957 | 99.54% |
| SWO_root_rep1 | 68,679,457 | 150 | 20,603,837,100 | 60.7 | SRR9831236 | 99.61% |
| SWO_root_rep2 | 61,446,391 | 150 | 18,433,917,300 | 60.7 | SRR9841186 | 99.60% |

**Supplementary Table 11. Summary of the bisulfite sequencing data for leaves of scion from SWO/SWO and SWO/Pt.**

| **Sample** | **Number of raw reads pairs** | **Read length (bp)** | **Total Bases (bp)** | **Mapping rates (%)** | **SRR ID** | **Conversion rate** |
| --- | --- | --- | --- | --- | --- | --- |
| SWO/SWO_rep1 | 65,469,376 | 150 | 19,640,812,800 | 71.2 | SRR9903452 | 99.51% |
| SWO/SWO_rep2 | 82,110,338 | 150 | 24,633,101,400 | 71.2 | SRR9903229 | 99.54% |
| SWO/Pt_rep1 | 86,640,142 | 150 | 25,992,042,600 | 70.5 | SRR9902303 | 99.54% |
| SWO/Pt_rep2 | 82,880,326 | 150 | 24,864,097,800 | 79.0 | SRR9912477 | 99.51% |

**Supplementary Table 12. List of the 1,537 genes that contain differentially methylated regions in their promoters between SWO/SWO and SWO/Pt.**

In separate EXCEL file.

**Supplementary Table 13. Kyoto Encyclopedia of Genes and Genomes (KEGG) pathway analysis of the 1,537 genes that contain differentially methylated regions in their promoters between SWO/SWO and SWO/Pt.**

In separate EXCEL file.

**Supplementary Table 14 Q-PCR analysis of differential methylated genes related to flavonoid biosynthesis and plant-pathogen interaction**.

| **Gene id** | **Relative expression level in**  **SWO/SWO** | **Relative expression level in**  **SWO/Pt** | **Gene description** |
| --- | --- | --- | --- |
| Cs1g_pb020830 | 1.00±0.05 | 1.53±0.07 | Flavanone 3-hydroxylase |
| Cs2g_pb023290 | 1.02±0.17 | 2.04±0.18 | Chalcone synthase |
| Cs4g_pb002370 | 1.00±0.04 | 27.3±0.60 | 4-coumarate-CoA |
| Cs4g_pb003610 | 1.00±0.03 | 5.45±0.19 | Cytochrome P450 |
| Cs5g_pb028920 | 1.00±0.02 | 5.52±0.22 | 4-coumarate--CoA ligase |
| Cs6g_pb003570 | 1.00±0.01 | 6.03±0.20 | Phenylalanine ammonia-lyase |
| Cs6g_pb003590 | 1.03±0.24 | 19.6±1.47 | Phenylalanine ammonia-lyase |
| Cs2g_pb025690 | 1.10±0.55 | 23.3±2.01 | Plant-pathogen interaction |
| Cs2g_pb026330 | 0.92±0.02 | 1.27±0.10 | Plant-pathogen interaction |
| Cs9g_pb013400 | 0.97±0.02 | 1.16±0.02 | Plant-pathogen interaction |

Data are mean ± s.d.; n = 3 technical replicates of 2 pooled tissue samples.

**Supplementary Table 15. Summary of small RNA sequencing data for the leaves from SWO/SWO and SWO/Pt.**

| **Sample** | **Clean reads** | **Unique reads** | **Mapping rate (%)** | **SRA accession** |
| --- | --- | --- | --- | --- |
| SWO_rep1 | 20406031 | 6029610 | 62.22 | SRR10829667 |
| SWO_rep2 | 35112248 | 8100729 | 57.35 | SRR10829824 |
| ZK_rep1 | 30395173 | 6559553 | 55.30 | SRR10829825 |
| ZK_rep2 | 36441252 | 7433684 | 56.16 | SRR10829826 |

**Supplementary Table 16. List of the 548 SWO/SWO-specific (RPM > 0 in SWO/SWO, not detected in SWO/Pt) and 356 SWO/SWO-highly expressed (≥ 1.5 fold) 24-nt siRNAs.**

In separate EXCEL file.

**Supplementary Table 17. Information of the primer sequences used for qPCR analysis of sRNA biogenesis related genes.**

| **Gene name** | **Primer (5'-3')** |
| --- | --- |
| **mRNA detection** |  |
| CLSY1 (Cs7g_pb025740) | F: TTGAAACCGCCCAGAGAGTA |
|  | R:CCAAGCAAAGGGTGTTGAAATA |
| DCL3 (Cs4g_pb005290) | F: CCGTGGTTAGAAAAGGCGTC |
|  | R:CTTTCTTTCGCTGAGGGGAC |
| NRPD1 (Cs5g_pb006000) | F: AAGCAATGCGGACCAGACTA |
|  | R:TGACAACATTAGGCACTCCAAA |
| RDR2 (Cs5g_pb011060) | F: ATGCTCTTTCCACAAGTATCCAG |
|  | R:AACATCTTCCGCACTCACCTC |
| SHH1 (Cs1g_pb015030) | F: GACACAGAAGGCTGCCAATG |
|  | R:GCGAATGAGGAAAAGAGGGA |
| **sRNA detection** |  |
| AAATGGATTAGGTATCCCATACCT | RT: GTCGTATCCAGTGCAGGGTCCGAGGTATTCGCACTGGATACGACAGGTAT |
|  | F: ATCCGAAATGGATTAGGTATCCC |
| ATACCAACATTCTTTTCCAAGATT | RT: GTCGTATCCAGTGCAGGGTCCGAGGTATTCGCACTGGATACGACAATCTT |
|  | F: ATCCGATACCAACATTCTTTTCC |
| AACTATTACGCCTATTGAGCGATC | RT: GTCGTATCCAGTGCAGGGTCCGAGGTATTCGCACTGGATACGACGATCGC |
|  | F: ATCCGAACTATTACGCCTATTGA |
| U6 | F: ACAGAGAAGATTAGCATGGCC |
|  | R:GACCAATTCTCGATTTGTGCG |
| Universal reverse primer | GTGCAGGGTCCGAGGT |

**Supplementary Table 18. Summary of the transcriptome sequencing data for *P. trifoliata* and sweet orange.**

| **Code** | **Number of raw reads pairs** | **Read length (bp)** | **Total Bases (bp)** | **Mapping rates (%)** | **SRR id** |
| --- | --- | --- | --- | --- | --- |
|
| Pt_shoot _rep1 | 30847309 | 150 | 9254192700 | 81.67 | SRR12589317 |
| Pt_shoot _rep2 | 28603879 | 150 | 8581163700 | 82.25 | SRR12589316 |
| Pt_shoot _rep3 | 30306189 | 150 | 9091856700 | 87 | SRR12589315 |
| Pt_root _rep1 | 25437958 | 150 | 7631387400 | 81.19 | SRR12595785 |
| Pt_root_rep2 | 30473975 | 150 | 9142192500 | 82.06 | SRR12595784 |
| Pt_root_rep3 | 29162722 | 150 | 8748816600 | 88.95 | SRR12595783 |
| SWO_shoot_rep1 | 25406877 | 150 | 7622063100 | 77.18 | SRR12587974 |
| SWO_shoot_rep2 | 26263271 | 150 | 7878981300 | 79.07 | SRR12587973 |
| SWO_shoot_rep3 | 39810978 | 150 | 11943293400 | 84.21 | SRR12587972 |
| SWO_root_rep1 | 25346505 | 150 | 7603951500 | 77.24 | SRR12587887 |
| SWO_root_rep2 | 31592731 | 150 | 9477819300 | 78.24 | SRR12587886 |
| SWO_root_rep3 | 36543882 | 150 | 10963164600 | 83.77 | SRR12587885 |

**Supplementary Table 19. Summary of small RNA sequencing data for *P. trifoliata* and sweet orange.**

| **Code** | **Clean reads** | **Unique reads** | **Mapping rates (%)** | **SRR id** |
| --- | --- | --- | --- | --- |
| Pt_shoot 1 | 50006044 | 13825855 | 68.29 | SRR12580663 |
| Pt_shoot 2 | 40047738 | 11902815 | 68.62 | SRR12580662 |
| Pt_shoot 3 | 47582112 | 12332681 | 68.30 | SRR12580661 |
| Pt_root 1 | 45851115 | 13678209 | 60.15 | SRR12580494 |
| Pt_root 2 | 44913376 | 13552878 | 61.38 | SRR12580493 |
| Pt_root 3 | 45764001 | 11955656 | 58.54 | SRR12580492 |
| SWO_shoot 1 | 31530376 | 8781042 | 63.50 | SRR12579744 |
| SWO_shoot 2 | 31772270 | 9408015 | 62.30 | SRR12579743 |
| SWO_shoot 3 | 54058087 | 13764429 | 61.35 | SRR12579742 |
| SWO_root 1 | 42289334 | 13881579 | 56.08 | SRR12579717 |
| SWO_root 2 | 42279201 | 13319267 | 52.13 | SRR12579716 |
| SWO_root 3 | 42385049 | 12104289 | 52.84 | SRR12579715 |
